# Reproductive development of common buckwheat (*Fagopyrum esculentum* Moench) and its wild relatives provides insights into their evolutionary biology

**DOI:** 10.3389/fpls.2022.1081981

**Published:** 2023-01-12

**Authors:** Dmitry D. Sokoloff, Raisa A. Malyshkina, Margarita V. Remizowa, Paula J. Rudall, Constantin I. Fomichev, Aleksey N. Fesenko, Ivan N. Fesenko, Maria D. Logacheva

**Affiliations:** ^1^Biological Faculty, Lomonosov Moscow State University, Moscow, Russia; ^2^Jodrell Laboratory, Royal Botanic Gardens, Kew, Richmond, United Kingdom; ^3^Buckwheat Breeding Lab, Federal Scientific Center of Legumes and Groats Crops, Orel, Russia; ^4^Center for Molecular and Cellular Biology, Skolkovo Institute of Science and Technology, Moscow, Russia

**Keywords:** bract, determinacy, *Fagopyrum*, flower, flowering unit, homology, inflorescence, Polygonaceae

## Abstract

**Introduction:**

Understanding the complex inflorescence architecture and developmental morphology of common buckwheat (*Fagopyrum esculentum*) is crucial for crop yield. However, most published descriptions of early flower and inflorescence development in Polygonaceae are based on light microscopy and often documented by line drawings. In *Fagopyrum* and many other Polygonaceae, an important inflorescence module is the thyrse, in which the primary axis never terminates in a flower and lateral cymes (monochasia) produce successively developing flowers of several orders. Each flower of a cyme is enclosed together with the next-order flower by a bilobed sheathing bract-like structure of controversial morphological nature.

**Methods:**

We explored patterns of flower structure and arrangement in buckwheat and its wild relatives, using comparative morphology, scanning electron microscopy and X-ray microtomography.

**Results:**

Our data support interpretation of the sheathing bract as two congenitally fused phyllomes (prophylls), one of which subtends a next-order flower. In *tepal-like bract*, a homeotic mutant of *F. esculentum*, the bilobed sheathing bract-like organ acquires tepal-like features and is sometimes replaced by two distinct phyllomes. Wild representatives of *F. esculentum* (ssp. *ancestrale*) and most cultivars of common buckwheat possess an indeterminate growth type with lateral thyrses produced successively on the primary inflorescence axis until cessation of growth. In contrast, determinate cultivars of *F. esculentum* develop a terminal thyrse after producing lateral thyrses. In contrast to *F. esculentum*, the occurrence of a terminal thyrse does not guarantee a determinate growth pattern in *F. tataricum*. The number of lateral thyrses produced before the terminal thyrse on the main axis of *F. tataricum* varies from zero to c. 19.

**Discussion:**

The nine stages of early flower development formally recognized here and our outline of basic terminology will facilitate more standardized and readily comparable descriptions in subsequent research on buckwheat biology. Non-trivial relative arrangements of tepals and bracteoles in *Fagopyrum* and some other Polygonaceae require investigation using refined approaches to mathematical modelling of flower development. Our data on inflorescence morphology and development suggest contrasting evolutionary patterns in the two main cultivated species of buckwheat, *F. esculentum* and *F. tataricum*. The genus *Fagopyrum* offers an excellent opportunity for evo-devo studies related to inflorescence architecture.

## Introduction

Cultivated species of the genus *Fagopyrum* include Common buckwheat (*Fagopyrum esculentum* Moench), the most economically important species of Polygonaceae, and *F. tataricum* (L.) Gaertn. (Campbell, 1997; Singh et al., 2020). Both species belong to the Cymosum group of *Fagopyrum*, which also includes the wild species *F. cymosum* (Trevir.) Meisn. and *F. homotropicum* Ohnishi, and has a centre of diversity in Sichuan, Yunnan and Tibet (Ohnishi and Matsuoka, 1996; Tsuji et al., 1999; Ohnishi, 2016; Zhou et al., 2018; Ohsako and Li, 2020). Molecular phylogenetic data support the monophyly of the Cymosum group and its sister relationship with the Urophyllum group, which comprises the remaining species of *Fagopyrum*. Morphologically, the Cymosum group is defined by broad cotyledons and fruits that greatly exceed the perianth in length (Ohnishi and Matsuoka, 1996; Ohnishi, 2016; Ohsako and Li, 2020). All four traditionally recognized species of the Cymosum group are morphologically well-defined. *Fagopyrum homotropicum* and *F. tataricum* are self-compatible with homostylous flowers whereas *F. cymosum* and *F. esculentum* are self-incompatible with distylous flowers (Ohnishi and Matsuoka, 1996; Fesenko et al., 2006; Chen et al., 2014; Ohsako and Li, 2020). *Fagopyrum cymosum* is a perennial herb that is sometimes utilised as a forage crop and also for medicinal purposes (Prakash and Yadav, 2016), whereas other three species are annuals. Apart from being self-compatible, *F. homotropicum* is morphologically closest to *F. esculentum*; the two species together forming a monophyletic unit (Ohnishi and Matsuoka, 1996; Ohnishi, 2016).

An agriculturally important feature of cultivated plants of both *F. tataricum* and *F. esculentum* is the absence of a constriction with an abscission layer on the pedicel, which prevents grain-shedding. Ancestrally wild accessions of *F. tataricum* (commonly referred as ssp. *potaninii*) and *F. esculentum* (commonly referred as subsp. *ancestrale*) share with *F. cymosum* and *F. homotropicum* the occurrence of articulate pedicels and shedding fruits (Ohnishi, 1990; 1998; Fesenko and Fesenko, 2015). *Fagopyrum tataricum* also includes weedy plants that apparently originated by hybridization between wild and cultivated lineages; some of these plants have non-articulated pedicels (Tsuji and Ohnishi, 2000). According to molecular data, *F. tataricum* is closely related to – and possibly derived from – *F. cymosum* (Yasui and Ohnishi, 1998; Yamane et al., 2003; Ohnishi, 2016). In seeds of *F. cymosum* and *F. tataricum*, the cotyledons are yellowish with transparent blade veins, whereas in *F. homotropicum* and *F. esculentum*, the cotyledons are colourless with opaque blade veins (Ohnishi and Matsuoka, 1996; Ohnishi, 2016).

A determinate growth pattern is crucial for crop yield in Common buckwheat, as in many other cultivated plants. It represents a functional trait of clear agricultural importance. Wild accessions and many cultivars of *F. esculentum* are indeterminate and their inflorescences maintain tip growth over a long period. Determinate cultivars of *F. esculentum* are characterized by inflorescences with more synchronous fruit maturation, which is an agriculturally important feature. Following detailed analysis by Fesenko (1968), the determinate morphotype of *F. esculentum* has been used successfully in breeding several commercialized cultivars that are currently widely available (Fesenko et al., 2018; Fesenko and Fesenko, 2019). The determinate growth pattern is inherited as a recessive homozygote in a yet unidentified locus designated locus “D” (Fesenko, 1968; Fesenko et al., 2010).

Based on different morphological grounds, determinacy has appeared in wild-type or mutant plants of various angiosperm species (e.g., Bradley et al., 1997; Singer et al., 1999; Teeri et al., 2006; Sinjushin, 2013; Benlloch et al., 2015; Saxena et al., 2017; Bommert and Whipple, 2018; Zhong and Kong, 2022). Mutations in the homologous genes *TERMINAL FLOWER 1* (*TFL1*) in *Arabidopsis* and *CENTRORADIALIS* in *Antirrhinum*, result in mutants with inflorescences that differ from those of the wild type in the presence of terminal flower. In *Pisum sativum* L. and some other legumes, mutations in homologues of *TFL1* result in the development of a terminal raceme (rather than a terminal flower), whereas the wild type has only lateral racemes (Benlloch et al., 2015). Furthermore, the (apparently genetically similar) transition to inflorescence determinacy in the Pigeon Pea (*Cajanus cajan* (L.) Huth) is realized through gain of a terminal double raceme, whereas the wild type has only lateral double racemes (Saxena et al., 2017).

Yurtseva (2006) provided an informative and comprehensive study of inflorescence diversity in Polygonaceae, with detailed discussion on organ homologies and evolutionary patterns, but she did not consider any member of *Fagopyrum* other than the indeterminate type of *F. esculentum*. Thus, despite the importance of the determinate cultivars of *F. esculentum*, they have not yet been characterized in detail morphologically. A study by Martynenko (1984) contained extensive quantitative data on both determinate and indeterminate material of *F. esculentum*, but use of these data is problematic because of uncertainty in the terminology adopted. To avoid repetition of this problem, the terminology used in this paper is summarized in Box 1. The use of basic descriptive terms is clarified with respect to buckwheat, in order to highlight the organ homologies. To date, no detailed SEM-based developmental data are available for inflorescences of any species of *Fagopyrum*, though Quinet et al. (2004) provided important data on inflorescence anatomy. The present study fills this knowledge gap by providing comparative developmental data on determinate and indeterminate cultivars of Common buckwheat. It seeks to place the inflorescence diversity of *F. esculentum* in a phylogenetic context by describing inflorescences of other members of the Cymosum group, plus *F. urophyllum* (Bureau & Franch.) Gross, an example of the Urophyllum group.

Inflorescences are composed of flowers, so inflorescence development cannot be evaluated in isolation from flower development. Bracts and bracteoles (floral prophylls) are key players in the interaction between flowers and inflorescences, the two primary hierarchical levels of plant reproductive architecture (Eichler, 1875; Prenner et al., 2009; Remizowa et al., 2013; Choob, 2022). Surprisingly, in contrast with many other cultivated plants and model angiosperms, buckwheat flower development has not been systematically characterized using SEM. Among the fragmentary SEM-based data on *Fagopyrum* flowers, Ronse De Craene and Smets (1991) studied the floral nectaries of *Fagopyrum*, Hong et al. (1998) investigated tepal surface morphology, and Logacheva et al. (2008) provided images of tepals and some other floral organs. However, most descriptions of early flower development in Polygonaceae are based on light microscopy and documented by line drawings (Payer, 1857; Schumann, 1890; Bauer, 1922; Galle, 1977), though late flower development in *Persicaria* was investigated using SEM (Ronse De Craene and Smets, 1994). A detailed study of flower development in *F. esculentum* was presented by Sattler (1973) using epi-illumination light microscopy, but this method does not allow precise documentation of all observations with fully convincing images. The present study provides complete SEM-based data on early flower development in *F. esculentum* and recognizes nine stages of early flower development that can be used for comparative purposes in subsequent experimental work.

In buckwheat and many other Polygonaceae (such as *Polygonum* and related genera), the homologies of the bilobed sheathing organ that is immediately associated with the flowers is controversial. Traditionally, the bilobed sheath was interpreted as a fusion product of two bracteoles, of which one subtends a next-order flower (Payer, 1857; Gross, 1913; Bauer, 1922; Galle, 1977; Ronse De Craene, 2022). However, Schumann (1890) questioned the developmental evidence for this interpretation. Yurtseva (2006) suggested that the sheath can be viewed as two fused stipules of one and the same prophyll and that a subtending bract of the next-order flower is absent in *Fagopyrum*. Among other arguments in favour of her theory, Yurtseva (2006) used observations on patterns of morphological variation in a homeotic mutant of *F. esculentum*, *tepal-like bract* (Fesenko et al., 2005; Logacheva et al., 2008). According to Kusnetzova et al. (1992), buckwheat has two bracteoles, whereas members of *Polygonum*, *Bistorta*, and *Persicaria* possess a single bracteole that (in contrast to the view of Yurtseva, 2006), subtends a next-order flower. We compare our developmental data on *Fagopyrum* with those on other Polygonaceae to the clarify morphological nature of the bilobed sheathing organ and reveal whether buckwheat differs in this respect from other members of the family.

To summarize, the present study was aimed in providing data on buckwheat structure and development at different hierarchical levels such as flower, cyme, thyrse and entire inflorescence, placing these data in a phylogenetic context and disentangling problems of organ homologies. The nine stages of early flower development recognized here and our outline of basic terminology will facilitate more standardized and readily comparable descriptions in subsequent research on buckwheat biology.

## Materials and methods

### Plant materials and treatments

We examined plant material of *F. esculentum*, *F. homotropicum*, *F. tataricum* and *F. urophyllum* cultivated in the Skolkovo Institute of Science and Technology (Moscow, Russia) and the All-Russia Research Institute of Grain Legumes and Groat Crops (Orel, Russia). At least 15 individual plants of each accession were examined. The following cultivars of *F. esculentum* subsp. *esculentum* were studied: Batyr, Chatyr Tau, Dasha, Dozhdik, and Temp. In addition, an accession of the wild subspecies *F. esculentum* subsp. *ancestrale* was examined (C9015, seeds received from the collection of Kyoto University, originally from Yongsheng district of Yunnan province, China) as well as an accession derived from its cross with cv. Dasha (FAD_F2_2018). Three accessions of *F. tataricum* were used: K17 is a cultivated line (received from the collection of Federal Research Center N.I. Vavilov All-Russian Institute of Plant Genetic Resources, St. Petersburg), C9119 is a wild accession from Lixian district of Sichuan province, China (received from Kyoto University) and Zhd001 comes from a seed collection made in a ruderal location near a railway in Moscow Province, Russia (55.587° N, 36.716° E). The accession of *F. homotropicum* examined here (С9139) comes from Yongsheng district of Yunnan province, China (received from Kyoto University). The accession of *F. urophyllum* (С9069) comes from southern China (received from Kyoto University).

Whole plants and young shoot tips were fixed in 70% ethanol. SEM work on material of the *tlb* mutant of *F. esculentum* was conducted at the Royal Botanic Garden, Kew (UK). The material was dissected in 70% ethanol, dehydrated through absolute ethanol and critical-point dried using an Autosamdri-815B CPD (Tousimis Research, Rockville, MD, USA). Material was mounted on SEM stubs, coated with platinum using an Emitech (Hailsham, East Sussex, UK) K550 sputter coater and examined using a Hitachi cold-field emission SEM S-4700-II (Hitachi, Japan). SEM work on material other than the *tlb* mutant was conducted at the Laboratory of Electron Microscopy at the Biological Faculty of Moscow University. The material was dissected in 70% ethanol and transferred to 100% acetone using the following series: 96% ethanol (twice for 30 min), 96% ethanol: 100% acetone (1:1 v/v, 30 min), 100% acetone (three times for 30 min). The material was critical-point dried using a Hitachi HCP-2 critical-point dryer (Hitachi, Japan), then coated with gold and palladium using a Eiko IB-3 ion-coater (Tokyo, Japan) and observed using a CamScan S-2 (Cambridge Instruments, London, UK) and a JSM-6380LA SEM (JEOL, Tokyo, Japan).

Specimens of *F. tataricum* (42 sheets) and *F. cymosum* (10 sheets) were examined in the Herbarium of Moscow University (MW) and the Herbarium of the Main Botanic Garden of the Russian Academy of Sciences, Moscow (MHA). In addition, available online resources of herbarium collections were examined. Inflorescence morphology of *F. urophyllum* was examined using the Chinese Virtual Herbarium (https://www.cvh.ac.cn/index.php). Fragments of herbarium material of *F. tataricum* were studied using high-resolution X-ray computed tomography (HRXCT). Dried herbarium material was rehydrated by soaking in boiling water and, transferred into an infiltration medium (1% phosphotungstic acid [PTA] in 70% ethanol) for a week to increase its contrast (Staedler et al., 2013). The PTA solution was exchanged four times. Then the samples were transferred in to 100% acetone using the following series: 1% PTA in 96% ethanol (twice for 30 min), 1% PTA in 96% ethanol: 100% acetone (1:1 v/v, 30 min), 1% PTA 100% acetone (three times for 30 min). The material was critical point-dried using a Hitachi HCP-2 critical point dryer (Hitachi, Japan) and then individually mounted to brass sample holder using carbon adhesive disc. The scans were performed on a SkyScan 1272 microtomograph (Bruker, Billerica, USA) equipped with a Hamamatsu L10101-67 source (Hamamatsu Photonics, Hamamatsu, Japan) and a Ximea xiRAY16 camera (Ximea GmbH, Münster, Germany) at the Biological Faculty of Moscow University. Source voltage and current were set to 35 kV and 175 μA, respectively, while exposure time was set to 550 ms, and no X-ray filter was used. The sample was rotated 180° around the vertical axis with a rotation step of 0.07. One tomographic acquisition was performed, resulting in an image stack of 2669 reconstructed micro-CT slices at 2 μm pixel size. Visualisation from the scanning data was performed using CTVox (Bruker, Billerica, USA). Images were manually coloured to show different phyllomes.

### Box 1. Terminology used in the present paper

Comparative and evolutionary studies of angiosperm inflorescence morphology are hindered by complexity and inconsistent use of descriptive terminology (Kusnetzova, 1988; Weberling, 1989; Bell and Bryan, 2008; Prenner et al., 2009; Endress, 2010). Unfortunately, available descriptions of inflorescence characters of buckwheat, either in taxonomic literature or publications on breeding, cannot be readily converted into homology-based terminology. Here, we attempt to clarify the use of basic descriptive terms with respect to buckwheat, in order to highlight the organ homologies.

#### Bract

This term lacks a uniform definition because its use is based on two features that are only partly overlapping across the seed plants, (1) a phyllome that bears in its axil a flower or an inflorescence module, or (2) a phyllome within an inflorescence that lacks a leaf lamina. Here, we use the term in the first sense, and always specify the nature of an axillary structure (i.e. flower-subtending bract or cyme-subtending bract).

#### Bracteose

Term describing an inflorescence (or its module) with subtending phyllomes on the main axis represented by scale-like leaves.

#### Cyme

An inflorescence module with a primary axis that is terminated in a flower and branching occuring only in the axil(s) of the prophyll(s). If there is only one branch, which is the case of *Fagopyrum*, then the cyme is a monochasium (Logacheva et al., 2008). By definition, a cyme can occupy only a lateral position in an entire inflorescence or plant body, because prophylls can only be recognized in a lateral axis. Some earlier studies described cymes as partial inflorescences (Fesenko et al., 2005; Fesenko and Fesenko, 2011), elementary inflorescences (Martynenko, 1984) or cymose lateral flowered clusters (Quinet et al., 2004), but the term cyme is much less ambiguous (used by 2007; 2009; Cawoy et al., 2006; Aubert et al., 2021).

#### Flowering unit

A module of the highest order that can be recognized in an inflorescence (more precisely, in a synflorescence). The primary axis has a terminal flowering unit. Paracladia, which are lateral axes of often more than one order, also terminate in flowering units, but the occurrence of lateral flowering units is not mandatory and may in part depend on environmental conditions. The term ‘flowering unit’ (Sell and Cremers, 1987; Vegetti and Anton, 2000; Acosta et al., 2009) is an English translation of the original French term ‘unite de floraison’ (Maresquelle, 1970; Sell, 1976). Kusnetzova (1988) proposed another translation, ‘floral unit’ (also adopted by Kostina and Yurtseva, 2021), which is less appropriate because other authors use the term in different ways (e.g., Tomlinson and Posluszny, 1978; Nitao and Zangerl, 1987; Claßen-Bockhoff and Bull-Hereñu, 2013). Some authors use the term ‘generative zone’ (‘zona plodoobrazovaniya’ in Russian) for the flowering unit of buckwheat (e.g. Martynenko, 1984; Fesenko et al., 2004; 2012).

#### Foliage leaf

Phyllome with a lamina.

#### Frondose

Inflorescence or its module in which the subtending the phyllomes on the main axis are represented by foliage leaves.

#### Frondo-bracteose

Inflorescence or its module in which the lower subtending phyllomes on the main axis are represented by foliage leaves and the upper subtending phyllomes by scale-like leaves.

#### Inflorescence

Part of the plant body bearing flowers. The term ‘inflorescence’ is ambiguous because different authors use different characters to draw a boundary between an inflorescence and the vegetative part of a plant. Synflorescence is a precisely defined term describing the flower-bearing region of the plant body that develops during a season. In many annuals such as Common buckwheat, the entire above-ground part of the body is a synflorescence.

#### Module

A repeating part of the plant body. Modules can be of several hierarchical levels. In inflorescences of *Fagopyrum*, these levels are flower, cyme, thyrse and flowering unit. The degree of similarity between modules varies. For example, *Fagopyrum* has both left- and right-handed flowers, whereas cymes may differ in the number of branching orders and are also left- and right-handed.

#### Paracladium (pl. paracladia)

A lateral axis terminated by an inflorescence module that repeats the module that terminates the entire inflorescence. In the present paper, we do not use the term paracladium for lateral thyrses (see Discussion).

#### Phyllome

General term for typical leaves and all their homologs (for example, foliage leaves, bud scales, bracts, sepals).

#### Prophyll(s)

Two (in many eudicots) or one (in many monocots) phyllomes that initiate the pattern of phyllotaxis on a lateral shoot; they can be analogized with cotyledon(s), which initiate the pattern of phyllotaxis of the primary axis of the entire plant (reviewed by Choob, 2022). Features in which prophyll(s) differ from the subsequent phyllomes of the lateral axis differ in various angiosperms and cannot be generalized. Prophylls associated with lateral flowers (floral prophylls) are alternatively called bracteoles.

#### Scale-like leaf

Phyllome lacking a lamina.

#### Subtending phyllome

One in which a lateral bud, shoot or flower is located in its axil (i.e., just above its level of insertion).

#### Thyrse

An inflorescence or inflorescence unit in which the primary axis never terminates in a flower and the lateral structures are cymes with successively developing flowers of two or more orders. The buckwheat thyrse is sometimes called simply ‘inflorescence’ (e.g., Quinet et al., 2004; Cawoy et al., 2006; 2007; 2009; Fesenko et al., 2010; Fesenko and Fesenko, 2014; Aubert et al., 2021). The term ‘raceme’ is morphologically incorrect for a thyrse.

## Results

### Thyrses, cymes and flowers: Structure and early development in *Fagopyrum esculentum*

**Figure 1:**
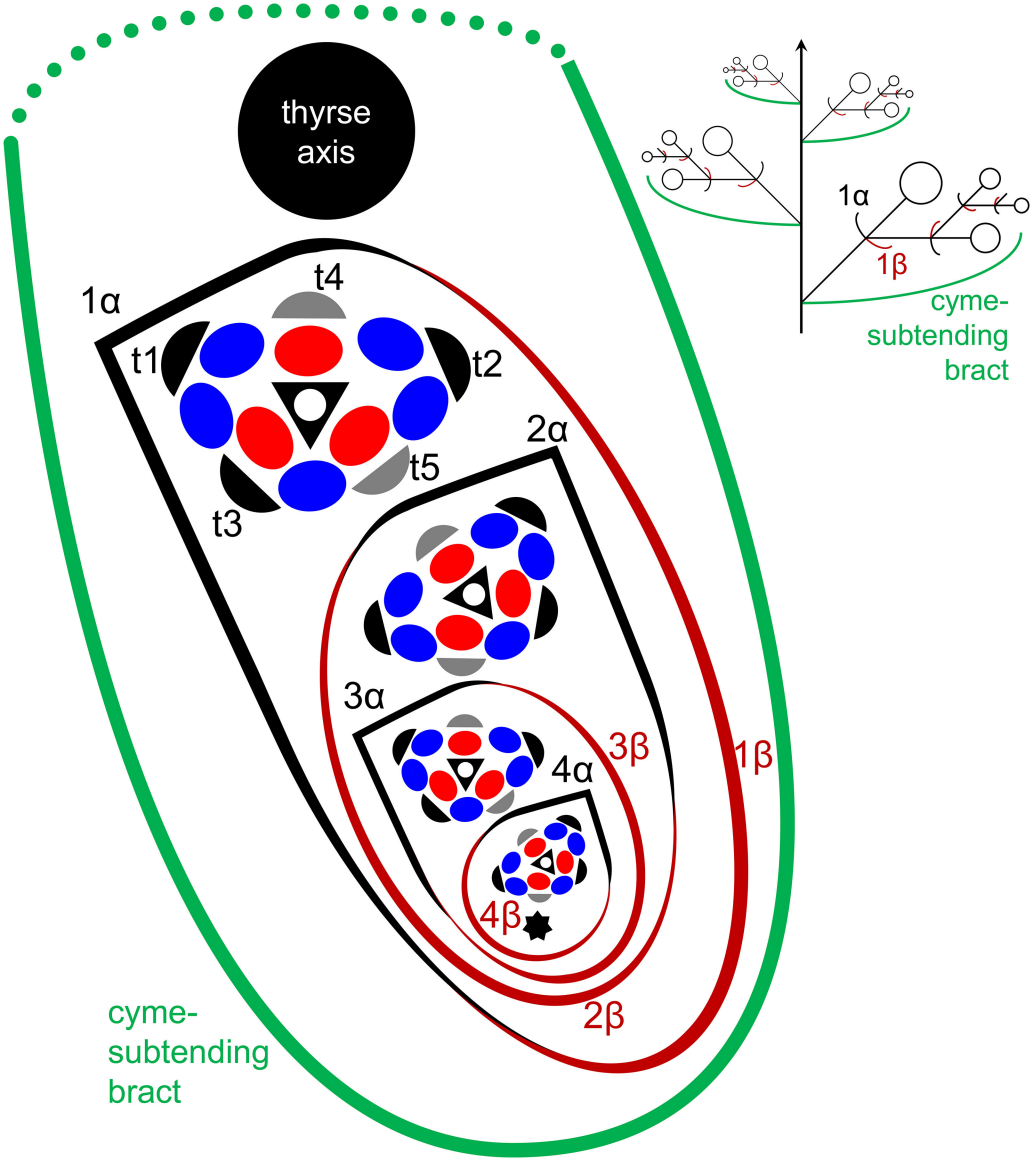
Flower, cyme and thyrse diagrams of *Fagopyrum esculentum.* Left, diagrammatic cross section of cyme with flowers of four successive orders and an incipient fifths flower (asterisk). α (black) and β (brown), two fused prophylls. Numbers before α and β indicate axis order within the cyme. t1-t5, tepals in in order of their inferred (pre)patterning. Two last-formed tepals are grey. Blue, outer whorl stamens; red, inner whorl stamens. Right, diagrammatic side view of thyrse with four cymes. Open circles, flowers. Cyme-subtending bract (green) is first narrow, but its base encircles the stem late in ontogeny (dotted line).

**Figure 2:**
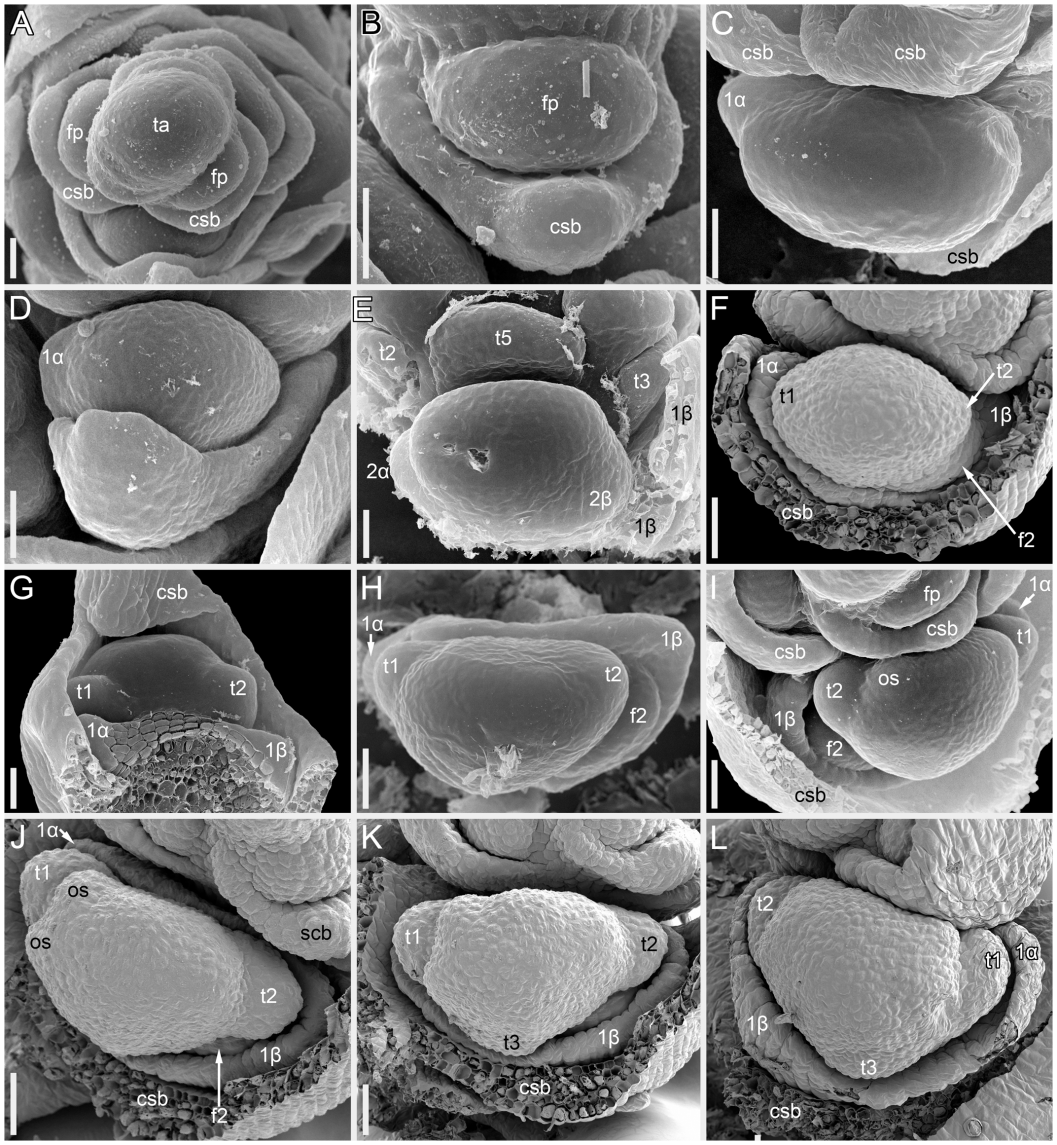
Flower development in *Fagopyrum esculentum*, stages 1–4 (SEM). **(A, B)** Stage 1. **(A)** Thyrse apex with spirally arranged cyme-subtending bracts and primordia of first flowers of cymes in their axils. **(B)** Primordium of the first flower of a cyme. **(C–E)** Stage 2. **(C, D)** The first flower of a cyme. **(E)** The second flower of a cyme. **(F–H)** Stage 3. **(F)** Cyme in abaxial view, cyme-subtending bract partly removed; early stage 3 with tepals 1 and 2 just slightly recognizable. **(G)** Cyme in adaxial view. **(H)** Cyme in top view, abaxial side down. **(I–L)** Stage 4. **(I, L)** Flowers that differ in handedness from the flower in **(J, K)**. **(J, K)** Different views of the same flower to show fusion of the two prophylls to form a tube (prophyllar sheath). **(A, E)** Chatyr Tau. **(B, C)** Temp. **(D)** Batyr. **(F, J–L)** Dasha. **(G)** Dozhdik. **(H, I)** FAD_F2_2018. 1α, 1β, prophylls of the first flower of a cyme; 2α, 2β, prophylls of the second flower of a cyme; csb, cyme-subtending bract; f2, the second flower of a cyme; fp, flower primordium; os, outer whorl stamen; t1-t5, tepals in in order of their inferred (pre)patterning; ta, thyrse apex. Scale bars = 30 µm **(A–L)**.

**Figure 3:**
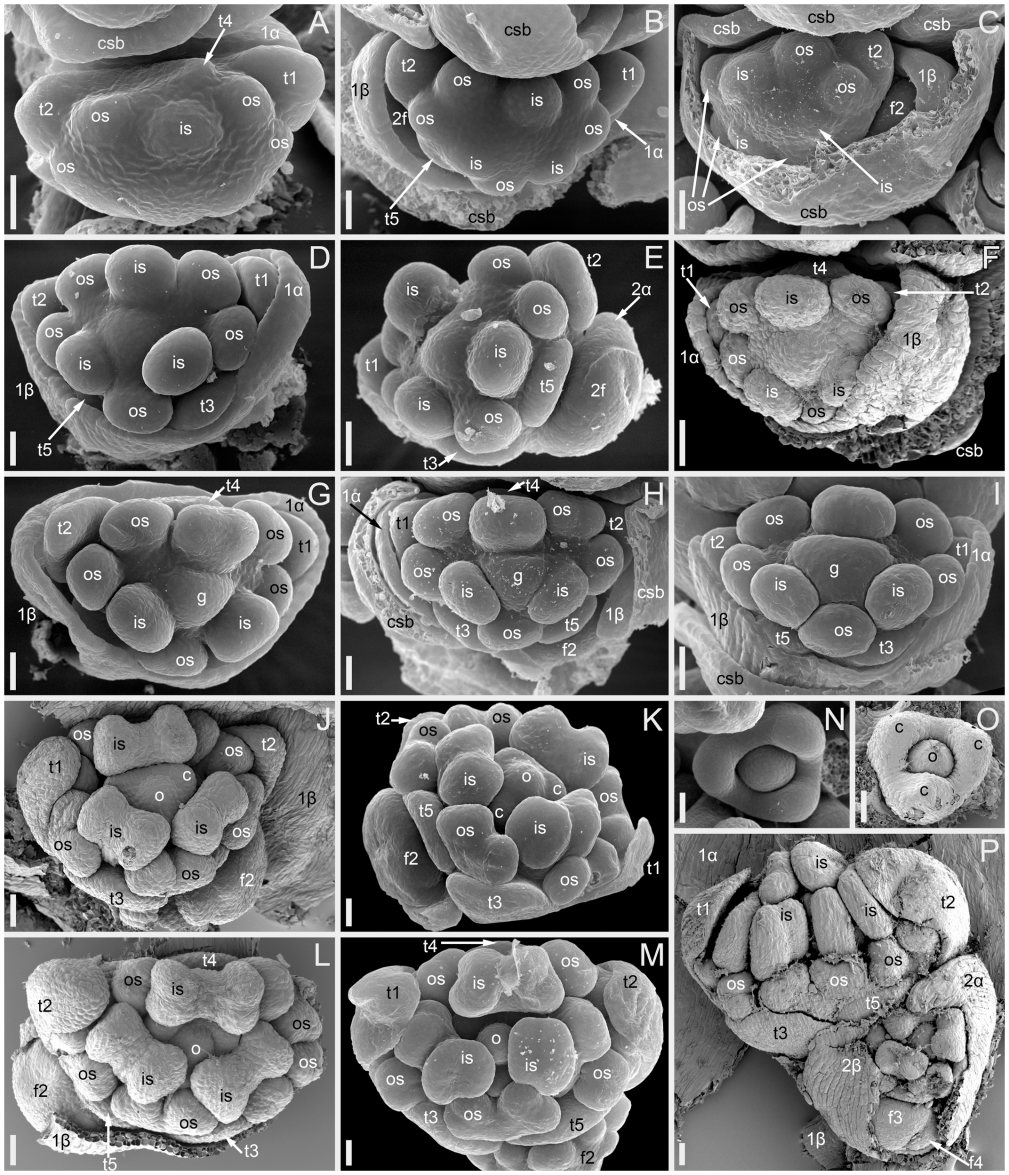
Flower development in *Fagopyrum esculentum*, stages 5–9 (SEM). **(A–C)** Stage 5. **(A)** Adaxial view of cyme with subtending bract removed and prophyllar sheath partly removed. Subtending bract of a younger cyme is visible and its imprint can be recognized between t2 and t4. The pressure made t4 asymmetric. **(B, C)** Two mirror-shaped cymes with subtending bracts partly removed. **(D–F)** Stage 6. The prophyllar sheath is removed in **(E)**. **(G–I)** Stage 7. The prophyllar sheath is partly dissected in **(H)**. **(J, K)** Stage 8. The prophyllar sheath is dissected in **(J)** and removed in **(K)**. **(L, M)** Stage 9. The prophyllar sheath is dissected in **(L)** and removed in **(M)**. **(J)** and **(M)** are mirror-shaped relative to **(K)** and **(L)**. **(N, O)** Gynoecia of flower in Stage 9. **(P)** Four-flowered cyme with flowers at successive developmental stages. The prophyllar sheath of the flower 2 is dissected. **(A, B, D, E, G, N)** FAD_F2_2018. **(C, H)** Temp. **(F, J, L, O, P)** Dasha. **(I, K, M)** Chatyr Tau. 1α, 1β, prophylls of the first flower of a cyme; 2α, 2β, prophylls of the second flower of a cyme; c, carpel apex; csb, cyme-subtending bract; f2, f3, f4, the second, the third and the fourth flower of a cyme; g, gynoecium; is, inner whorl stamen; o, ovule; os, outer whorl stamen; t1-t5, tepals in in order of their inferred (pre)patterning. Scale bars = 30 µm **(A–P)**.

In *Fagopyrum* and many other Polygonaceae, the thyrse represents an important inflorescence module. In a thyrse, the primary axis never terminates in a flower and there are lateral cymes with successively developing flowers of several orders. Each cyme is located in the axil of a cyme-subtending bract, which is also the subtending bract of the first flower (**Figure 1**). With very few exceptions (see below), cyme-subtending bracts are scale-like green phyllomes with no traces of even a reduced leaf lamina. Cyme-subtending bracts are spirally arranged along the thyrse axis (**Figure 2A**). Thyrses with clockwise and anticlockwise spirals are both found, without any noticeable regularity. Each flower of a cyme is enclosed together with the next-order flower by a thin, membranous and transparent bilobed sheath interpreted here as two congenitally fused prophylls (α-prophyll and β-prophyll). We use the term ‘prophyllar sheath’ for this structure. The β-prophyll is a subtending bract of the next-order flower of the cyme (**Figure 1**). The position of the next-order flower is intermediate between transversal and abaxial relative to the cyme-subtending bract (i.e. it is closer to the subtending bract than to the thyrse axis). The next-order flower and its prophyllar sheath repeat the morphology of those of the first order but are mirror-shaped. There is a change of handedness with every next order of branching in the cyme. As a result, successive flowers of a cyme form a zig-zag pattern (**Figures 1**, **3P**). The cyme of *Fagopyrum* can be classified as a monochasium because branching always takes place in the axil of a single prophyll, and more precisely as a cincinnus because of the zig-zag pattern of flower arrangement. Two mirror-shaped types of cincinni can be recognized that differ in the left- or right-hand positions of the α- and β-prophylls of the first flower and consequently of the second flower. For example, the β-prophyll of the first flower and the second flower situated in its axil are in right-hand positions in **Figures 1**, **2J, K**, **3C** and in left-hand positions in **Figures 2I**, **3B**.

Flowers of *Fagopyrum* share with several other Polygonaceae the occurrence of five tepals, five outer-whorl stamens, three inner-whorl stamens and three united carpels (**Figure 1**). There is a single ovule that is attached at the base of the unilocular ovary and shared by all three carpels. The tepals can be numbered according to their inferred sequence of (pre)patterning, though the sequence of their actual appearance is very rapid. This numbering agrees with tepal aestivation (i.e., the mode of their overlapping) that can be seen at the latest developmental stages (not shown). Tepals 1 and 2 are in transversal positions. Tepal 1 is in the same radius as the α-prophyll (**Figure 1**). Tepal 4 is in the adaxial (=posterior) part of the flower (the part that is closer to the thyrse axis or to the previous flower of the cyme). Tepals 3 and 5 are in the abaxial (anterior) part of the flower (the part that is closer to the subtending bract). Tepal 3 is closer to tepal 1 and tepal 5 is closer to tepal 2 (**Figure 1**). The next-order flower is located in the sector of tepal 5 (**Figures 1**, **3E, H, J, K, M**). The five outer-whorl stamens could be viewed as alternating with the five tepals, but they are not equally spaced early in development. Two stamens adjacent to tepal 1 are closely spaced and form a pair. Similarly, there is another pair of stamens adjacent to tepal 2 (**Figures 1**, **3D, E, G-I**). Of the three inner-whorl stamens, one is adaxial and two are transversal-abaxial. The inner-whorl stamens alternate in radii with the three carpels, of which one is abaxial and two are adaxial-transversal.

The following stages of early flower development can be recognized in *F. esculentum*. We found no differences in flower development among observed cultivars of the Common buckwheat (Dasha, Temp, Chatyr Tau, Batyr, Dozhdik) and its accession FAD_F2_2018.

Stage 1 (**Figures 2A, B**). Flower primordium. The primordium is elliptic in outline. There is no evidence of prophyll or tepal initiation at this stage.

Stage 2 (**Figures 2C–E**). Prophyll(s) are initiated, but tepals cannot be recognized. It is likely that the α-prophyll initiates before the β-prophyll, but **Figures 2C, D** cannot be taken as robust proof of this statement, because the β-prophyll may be hidden in these views.

Stage 3 (**Figures 2F–H**). The two prophylls are clearly united into a sheathing tube by congenital fusion. Tepals 1 and 2 are initiated. There is no evidence of stamen initiation at this stage.

Stage 4 (**Figures 2I–L**). Tepal 3 is initiated. Outer-whorl stamens initiate in pairs (sometimes as common primordia)? in sectors of tepals 1 and 2. The fifth outer-whorl stamen cannot be traced at this stage.

Stage 5 (**Figures 3A–C**). All tepals and all outer-whorl stamens are initiated. Inner-whorl stamens are present, though one of them may be yet weakly defined (the one that is closer to the next-order flower, **Figure 3C**). Therefore, it is likely that the inner-whorl stamens initiate in a rapid sequence. No evidence of gynoecium initiation so far.

Stage 6 (**Figures 3D–F**). The gynoecium can be recognized as a dome-shaped primordium between the three inner-whorl stamens. Stamens, especially those of the inner whorl, are larger than in Stage 5, but thecal structure is not yet pronounced. At this stage, the next-order flower cannot be observed without dissection of the prophyllar sheath.

Stage 7 (**Figures 3G–I**). The gynoecium is triangular in top view. Thecal structure can be recognized at least in the median-adaxial inner-whorl stamen (the only stamen unlabelled in **Figures 3G–I**).

Stage 8 (**Figures 3J, K**). The ovule can be seen at this stage, surrounded by the shallow rim of the gynoecium wall with the tips of the three carpels. Thecae are well-pronounced in all stamens. The inner-whorl stamens are longer than the outer-whorl stamens and possess short filaments. Their anther bases are below the level of the gynoecium rim.

Stage 9 (**Figures 3L–O**). The gynoecium rim has increased in height and the carpel tips are facing upwards and exceed the ovule. The inner-whorl stamens much exceed the outer-whorl ones. By filament elongation, their anthers extend above the gynoecium.

### Cyme structure and flower development in the *tepal-like bract* mutant of *Fagopyrum esculentum*

**Figure 4:**
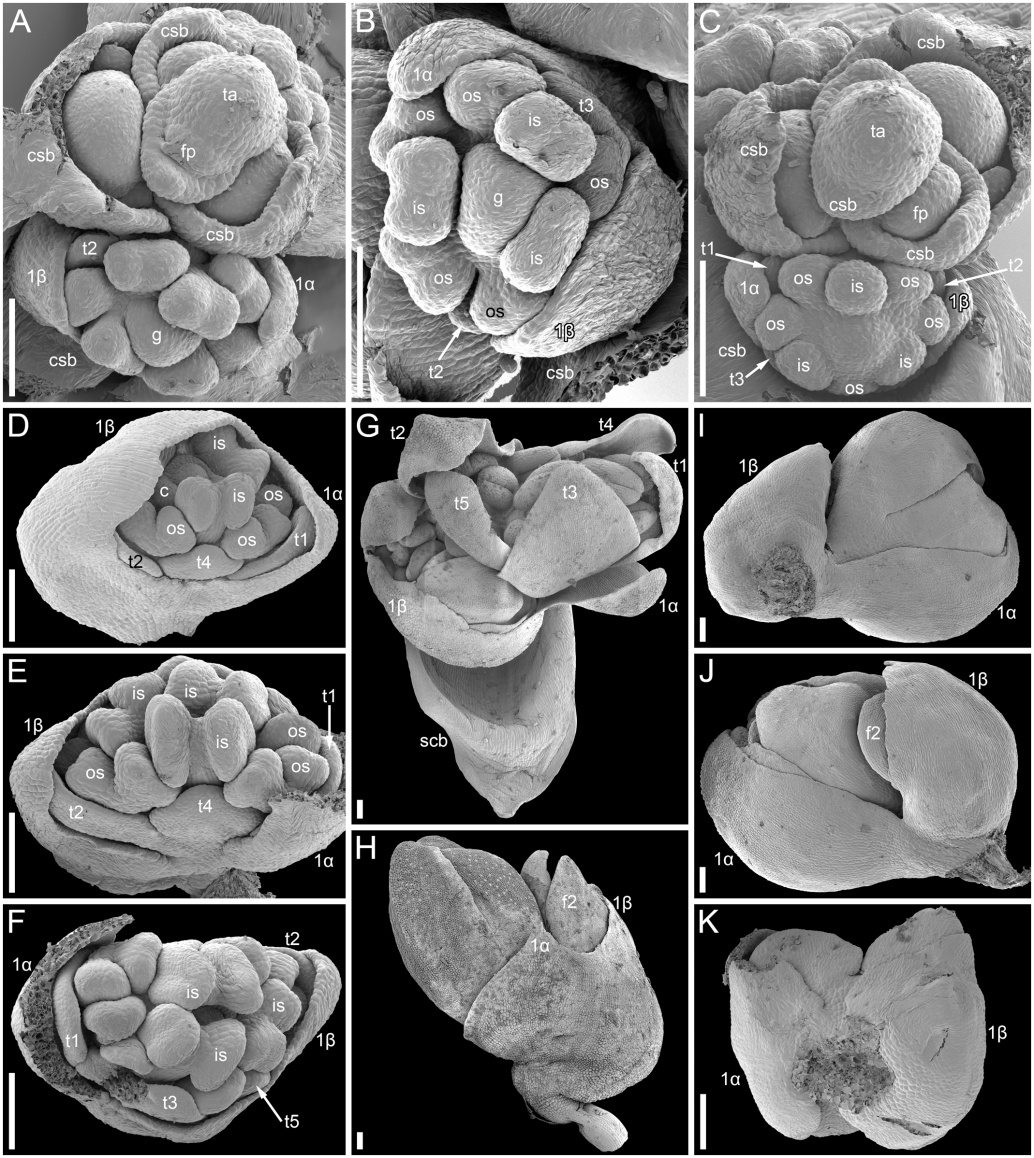
Flower development in the *tepal-like bract* mutant of *Fagopyrum esculentum* (SEM). **(A–C)** Images taken from different sides of a young thyrse with flowers at successive developmental stages. **(B)** The oldest cyme of the specimen. The same cyme is shown in the lower part of **(A)**. The first flower of this cyme is at Stage 7 (note the triangular gynoecium with no evidence of ovule initiation as well the occurrence of thecae in some stamens). The α and β prophylls are largely free (or only basally united). **(C)** View showing cyme adjacent to that in **(B)**, also visible in the upper right part of **(A)**. The first flower of this cyme is at Stage 6 (the gynoecium is dome-shaped rather than triangular, stamen thecae not yet initiated). **(D)**. Flower at Stage 8 (the ovule is initiated, but anthers of inner stamens are shorter than at Stage 9). The α and β prophylls are fused to form a pronounced tube. **(E, F)** Two views of a flower at Stage 9 (anthers of the inner whorl stamens exceed the gynoecium). The α and β prophylls are united on the anterior side of the flower **(F)**, but free on the posterior side **(E)**. **(G)** Cyme similar to that in **(E, F)**, but at a later developmental stage. **(H–J)** Cymes with closed mature floral buds. **(H)** The prophyllar sheath is well-pronounced. **(I, J)** Two views of a cyme with the α and β prophylls are only basally united. **(K)** Cyme with completely free prophylls. 1α, 1β, prophylls of the first flower of a cyme; c, carpel apex; csb, cyme-subtending bract; f2, the second flower of a cyme; g, gynoecium; is, inner whorl stamen; os, outer whorl stamen; t1-t5, tepals in in order of their inferred (pre)patterning; thyrse apex. Scale bars = 100 µm **(A–K)**.

The mutant differs from the wild type in the tepal-like nature of the cyme prophylls. In particular, conical cells can be found on the abaxial surface of the prophylls. However, expression of the tepal-like features is unstable; in the cyme illustrated in **Figure 4J**, the α prophyll has conical cells but the β prophyll lacks them. The two prophylls that are always united to form a sheath in the wild type tend to be separate in the mutant, though the degree of separation varies; the two prophylls can be completely free from each other (**Figure 4K**), very shortly united at both sides (**Figures 4I, J**), united at the abaxial side only (**Figures 4E, F**) or united to form a proper sheath (**Figure 4D**). There is a tendency for elongation of axes of all orders below and between the prophylls. When the prophylls are united, this causes the cyme base to be crooked (**Figure 4H**). Our data on early flower development showed great similarity to the wild type (**Figures 4A-F**). Organ number and their positions are the same as in the wild type. Developmental data suggest the sporadic development of the α and β prophylls as two free organs rather than the sporadic appearance of yet another phyllome in the cymes. The two almost-free phyllomes visible in **Figure 4B** can be readily identified as α and β prophylls.

### Inflorescence morphology in the Cymosum group of *Fagopyrum*

All species examined possessed thyrses of similar structure as described above for *F. esculentum.* Cyme-subtending phyllomes were scale-like, unless directly specified below.

#### Fagopyrum cymosum

**Figure 5:**
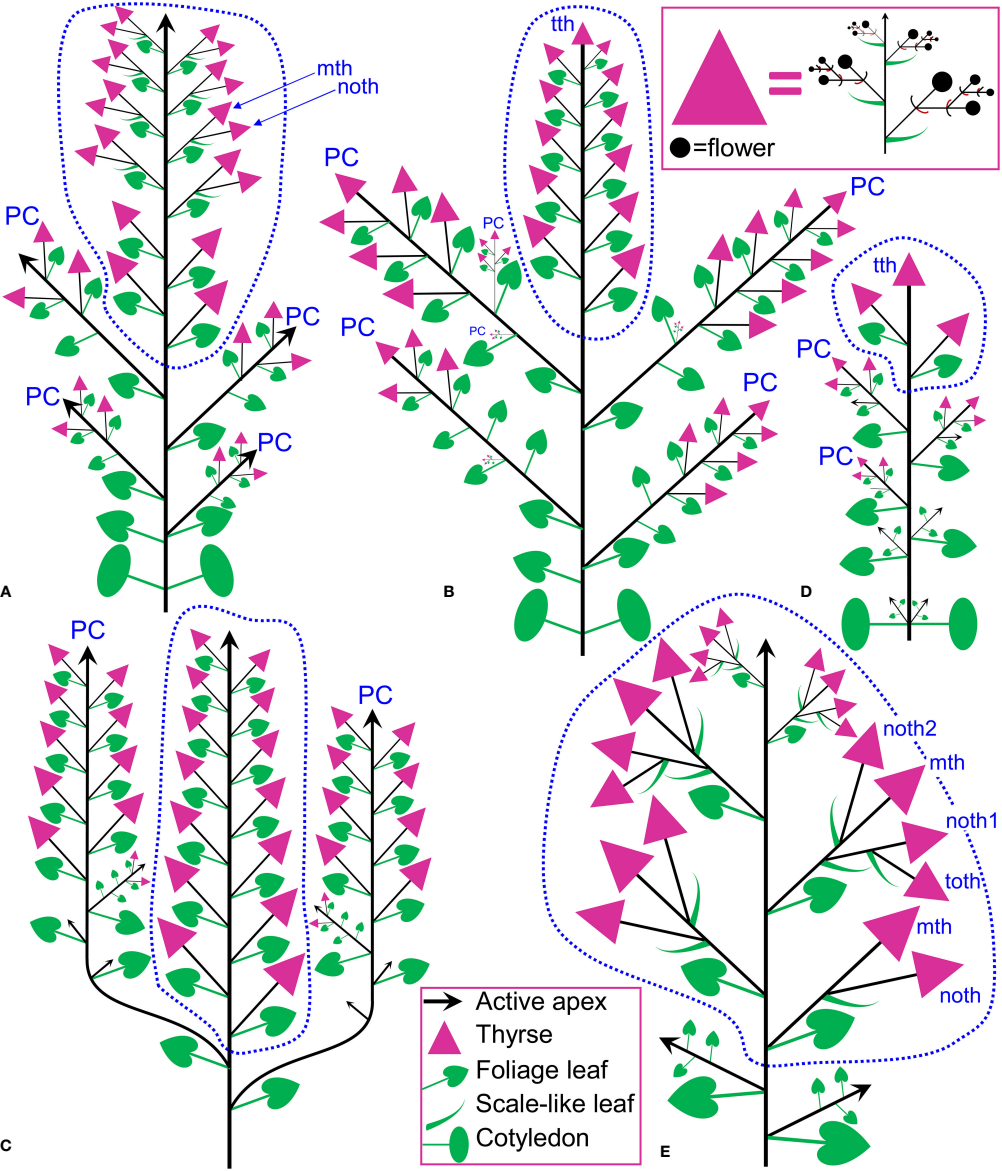
Inflorescence diagrams. **(A)** *Fagopyrum tataricum*, accession K17, entire plant cultivated in the same conditions as the plant in **(B)** and examined at a similar developmental stage (measured by the number of leaves on the main axis). With subsequent growth, the plant will ultimately produce a terminal thyrse. **(B)** *Fagopyrum tataricum*, accession C9119, entire plant illustrated **Figure 7**. **(C)** *Fagopyrum esculentum* subsp. *ancestrale*, upper part of inflorescence. There are more paracladia of similar structure, but shorter than the two illustrated here, in axils of lower leaves of the main axis. Such paracladia are present in axils of cotyledons, too. **(D)** *Fagopyrum esculentum* subsp. *esculentum* cv. Dozhdik, entire plant. **(E)** *Fagopyrum cymosum*, upper part of shoot system based on the herbarium specimen illustrated in **Figure 6A**, **(B)** Dashed line, terminal flowering unit; mth, main thyrse of a lateral group; noth, next order thyrse of a lateral group; PC, paracladium; toth, third order thyrse of a lateral group; tth, terminal thyrse.

**Figure 6:**
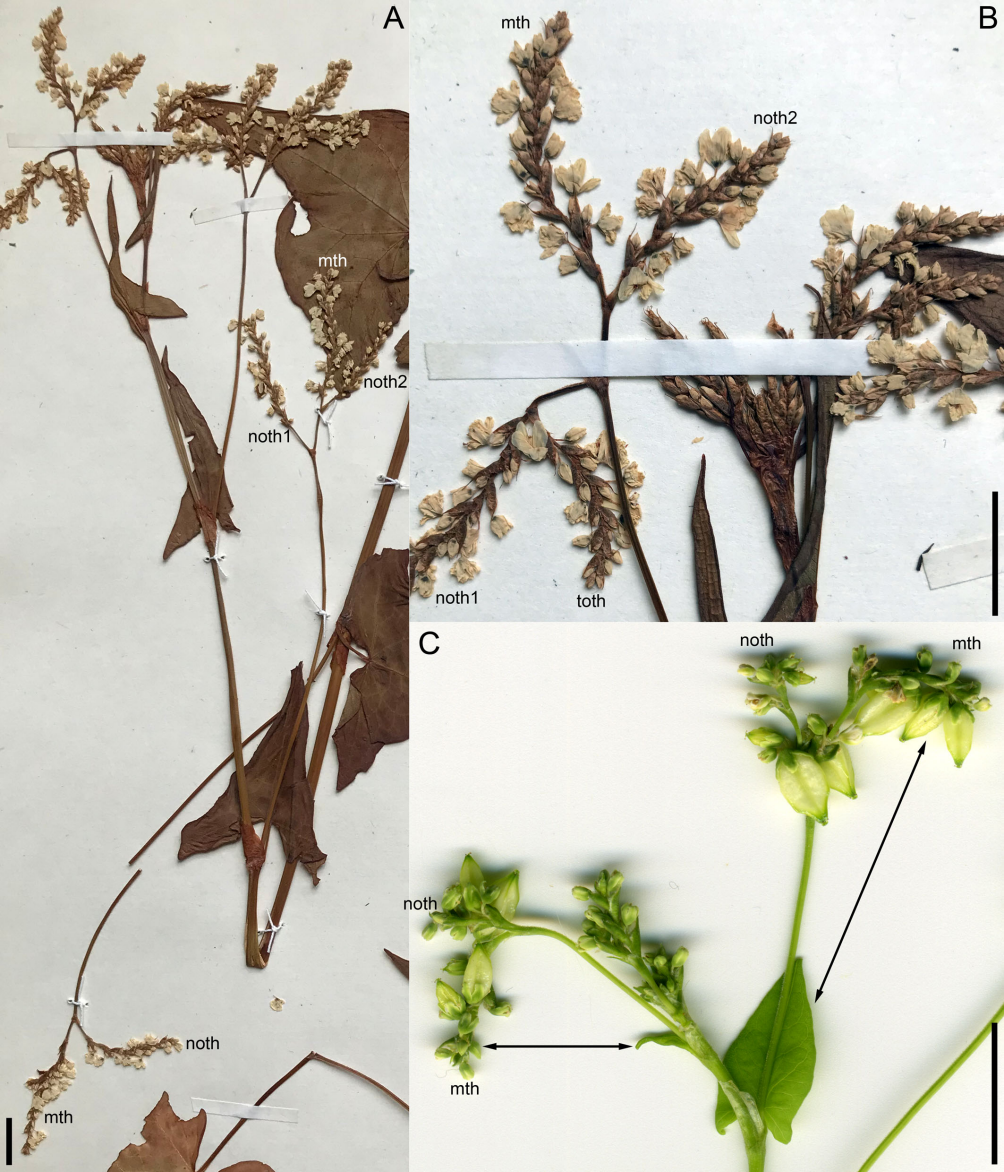
Inflorescence morphology. **(A, B)** *Fagopyrum cymosum* (herbarium specimen: China, Yunnan, *Bartholomew et al., Sino-Amer. Bot. Exped. No. 1214 –* MHA). **(B)** is a detail of **(A)** showing young branches near the apex of the main axis. A diagram of this specimen is in the **Figure 5E**. **(C)** *Fagopyrum tataricum* accession K17, distal part of inflorescence. See **Figure 5A**, for complete diagram. Arrows show leaves subtending particular thyrses. mth, main thyrse of a lateral group; noth, next order thyrse of a lateral group; toth, third order thyrse of a lateral group; tth, terminal thyrse. Scale bars = 1 cm **(A–C)**.

This species belongs to the same clade as the two main cultivated species, *F. tataricum* (**Figures 5A, B**) and *F. esculentum* (**Figures 5C, D**), but differs in being a perennial. Our inflorescence diagram of *F. cymosum* (**Figure 5E**) is based on a specimen whose distal part is illustrated in **Figures 6A, B**. The main axis of the inflorescence is not terminated by a thyrse (**Figures 5E**, **6B**), at least at developmental stages available in collections. The main axis bears only foliage leaves (**Figures 5E**, **6A**). Inflorescence branches located in the axils of these foliage leaves bear only scale-like leaves; in each branch, the next-order branch(es) have thyrse(s) and are themselves terminated in a thyrse (**Figures 5E**, **6A**). The lowermost branch has one next-order thyrse, the second branch has two next-order thyrses and subsequent branches have a first-order thyrse, two second-order thyrses and a third-order thyrse (**Figure 5E**). The lowermost branches appear vegetative in this particular specimen (**Figure 5E**), but analysis of other specimens showed that such branches can develop as paracladia, repeating the branching pattern of the distal part of the main axis.

A few collections of *F. cymosum* (e.g., P04961395, Muséum national d’Histoire naturelle, Paris) possess inflorescences similar to that of **Figure 5E**, but with even more extensive branching of lateral axes bearing thyrses. These branches can be described as panicles of thyrses. As in all other collections of *F. cymosum*, only the main inflorescence axis bears foliage leaves and does not produce a terminal thyrse. Lateral axes of all orders bear only scale-like leaves and are all terminated in thyrses.

#### Fagopyrum tataricum

**Figure 7:**
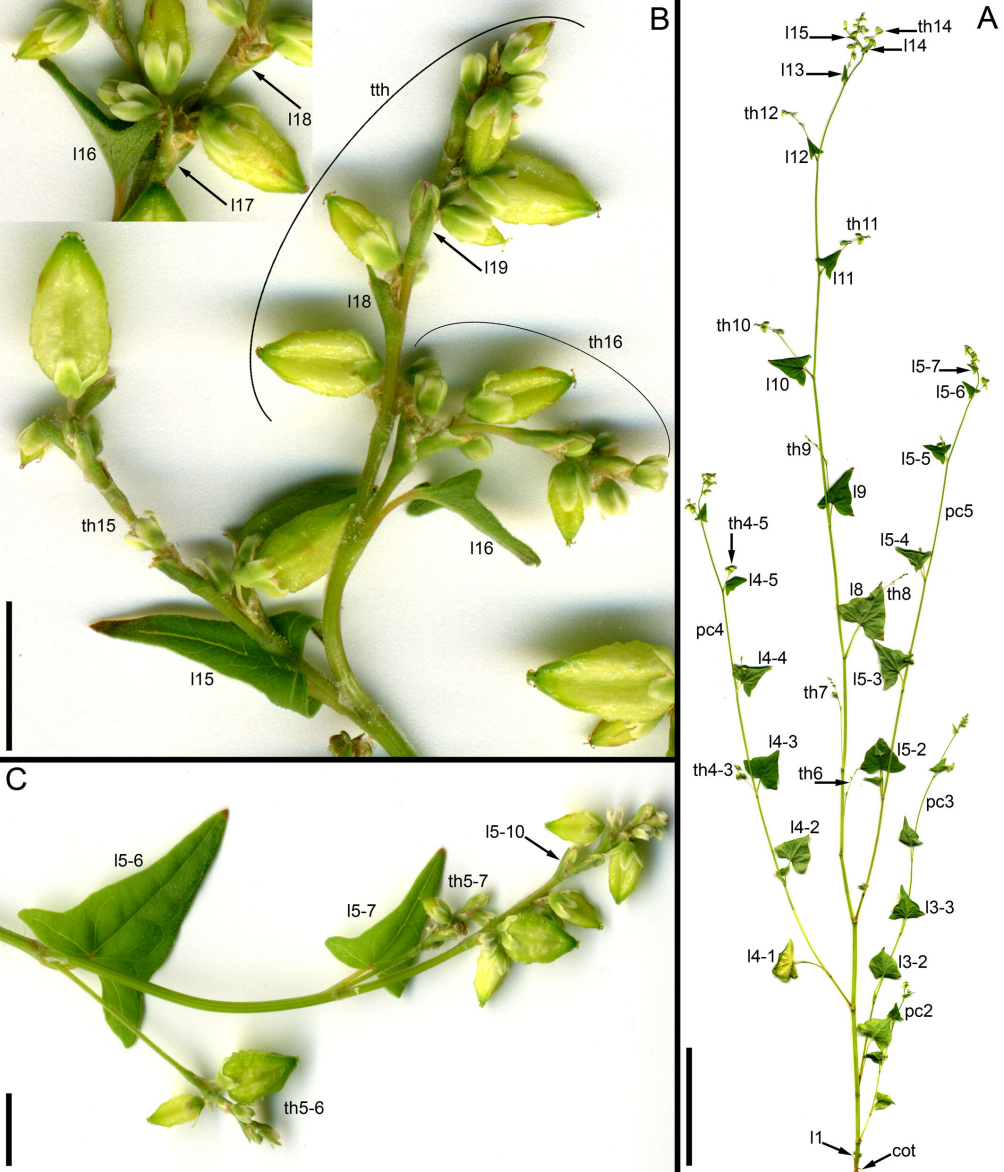
Morphology of *Fagopyrum tataricum*, accession C9119. **(A)** Entire plant. Its diagram is provided in **Figure 5B**. **(B)** Detail showing a terminal thyrse of the terminal flowering unit and two uppermost lateral thyrses. **(C)** Detail showing a terminal thyrse of the uppermost paracladium and two lateral thyrses below it. cot, cotyledonary node; l, leaf (foliage leaf or cyme-subtending bract); pc, paracladium; th, lateral thyrse; tth, terminal thyrse. Leaves of the main axis are numbered (cotyledons not counted). Leaves of lateral axes have double numbers, the first one being the number of the main axis leaf subtending the branch. Numbers of axillary paracladia and thyrses are those of their subtending leaves. Scale bars = 10 cm **(A)**, 5 mm **(B, C)**.

This is an annual species that comprises both cultivated and wild accessions. Wild plants of *F. tataricum* from Southeast Asia are known as subsp. *potanini*, but apparently this name is not formally published. In addition, *F. tataricum* is widely distributed across temperate Eurasia as ruderal plant. We examined in detail living material of three accessions, K17 (cultivated, **Figures 5A**, **6C**), C9119 (wild accession of ‘subsp. *potanini*’, **Figures 5B**, **7**) and Zhd001 (ruderal plant, **Supplementary Figure 1**) as well as available herbarium specimens. In accession K17 (**Figure 5A**), the terminal flowering unit (outlined by a dotted blue line in **Figure 5A**) first develops lateral bracteose thyrses (four thyrses in **Figure 5A**). In the middle region of the flowering unit, each foliage leaf of the main axis subtends a lateral thyrse supplemented by a next order thyrse developing in the axil of a scale-like phyllome of the same morphology as cyme-subtending bracts (**Figure 5A**). At the stage when c. 12 nodes of the flowering unit are formed (**Figure 5A**), the absence of a thyrse terminating the entire inflorescence was a remarkable and stable difference from the accession C9119 in our common garden experiment. However, when c. 19 nodes of the flowering unit were formed and lower lateral thyrses already became postanthetic, the main axis of K17 ultimately developed a terminal thyrse. Below the terminal flowering unit, the main axis possesses foliage leaves with axillary paracladia, which develop smaller flowering units that are delayed in development relative to the terminal flowering unit. Upper paracladia are larger than lower ones. We found herbarium specimens of cultivated *F. tataricum* with inflorescence morphology fitting the pattern described here for the accession K17 (e.g., P04619572, Muséum national d’Histoire naturelle, Paris). These herbarium samples showed no thyrse terminating the flowering unit, but one cannot rule out its late appearance by analogy with our sample K17.

In accession C9119, the main inflorescence axis is terminated by a thyrse at an earlier stage than that of K17 (**Figures 5B**, **7B**). About ten nodes of the main axis below the terminal thyrse bear foliage leaves with axillary thyrses. Their development, anthesis and fruit maturation are acropetal. The terminal and lateral thyrses form a terminal flowering unit (dotted blue line in **Figure 5B**). Foliage leaves on the main axis situated below the terminal flowering unit possess axillary paracladia that develop smaller flowering units of the same principal architecture. In particular, these lateral flowering units also possess terminal thyrses (**Figures 5B**, **7C**). Second-order paracladia can be also found (**Figure 5B**), but they are strongly delayed in development.

**Figure 8:**
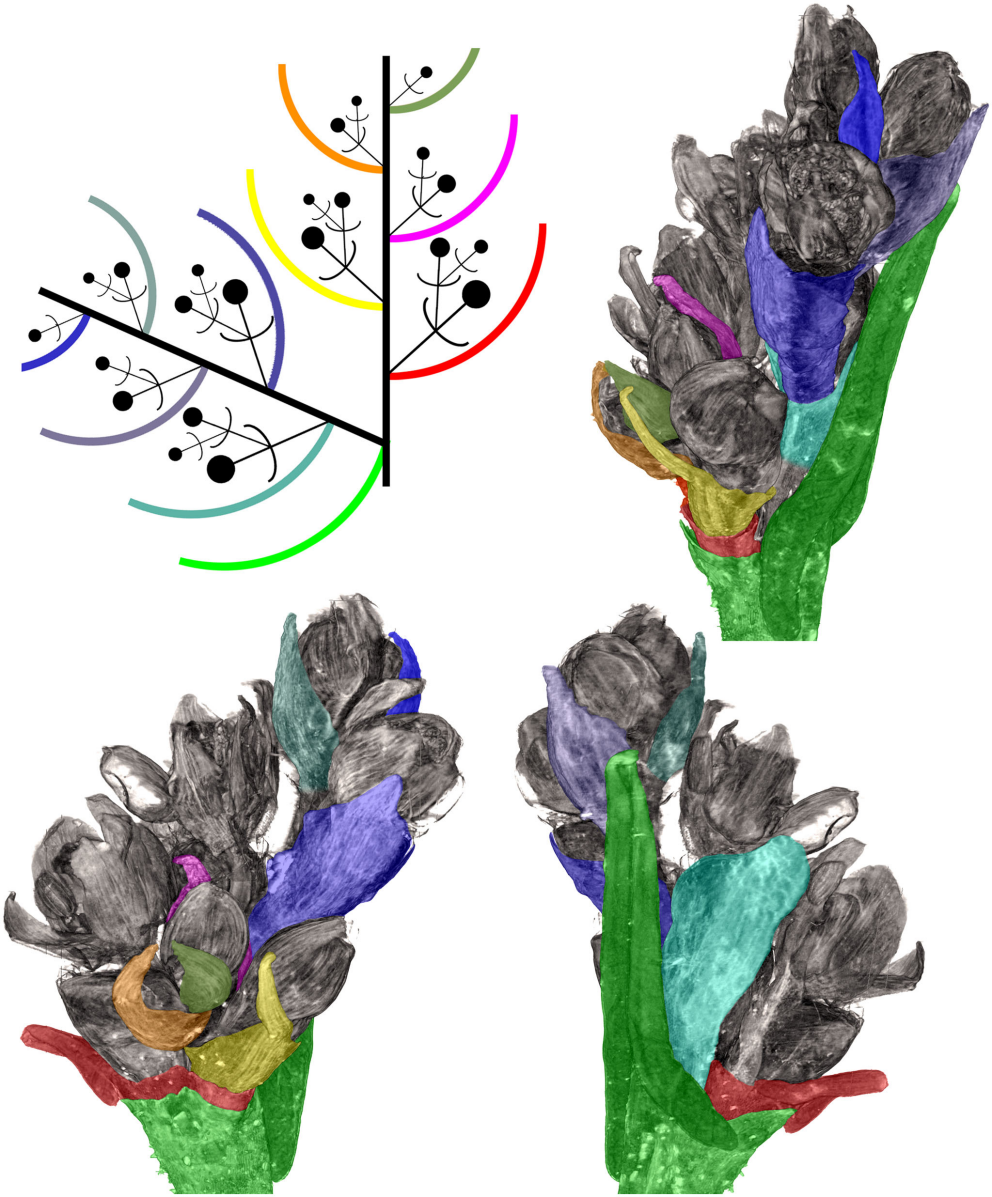
Distal part of flowering unit in a herbarium specimen of a ruderal plant of *Fagopyrum tataricum* from Middle Russia (MW0325480). Different views of a 3D image produced using X-ray microtomography and manually coloured to show different organs. A diagram in the upper left corner can be used as key to colours. The uppermost lateral thyrse and terminal thyrse are shown.

Examined herbarium specimens of ruderal plants of *F. tataricum* from Russia and Mongolia, where it was possible to uncover details of branching, possessed inflorescences similar to those in C9119, but there was variation in the structure of the terminal thyrses. Some accessions possessed a small and developmentally delayed terminal thyrse with proximal cyme-subtending phyllomes resembling young foliage leaves subtending lateral thyrses in possessing incepted laminas (**Figure 8**). Other accessions (e.g., MW0175953, Herbarium of Moscow University), instead possessed long terminal thyrses, often with long internodes between proximal cyme-subtending phyllomes. The proximal cyme-subtending phyllomes were sometimes differentiated as foliage leaves and the thyrse apex was apparently active for a long period producing young cymes. In the smallest of the tree individuals of the herbarium specimen MW0175953, the entire inflorescence is composed of a terminal thyrse. All plants of the accession Zhd001 grown in our experiment possessed this morphology and developed an inflorescence composed of a terminal frondo-bracteose thyrse. The third leaf above the cotyledons already subtended a cyme in these plants (**Supplementary Figure 1**).

#### Fagopyrum esculentum

**Figure 9:**
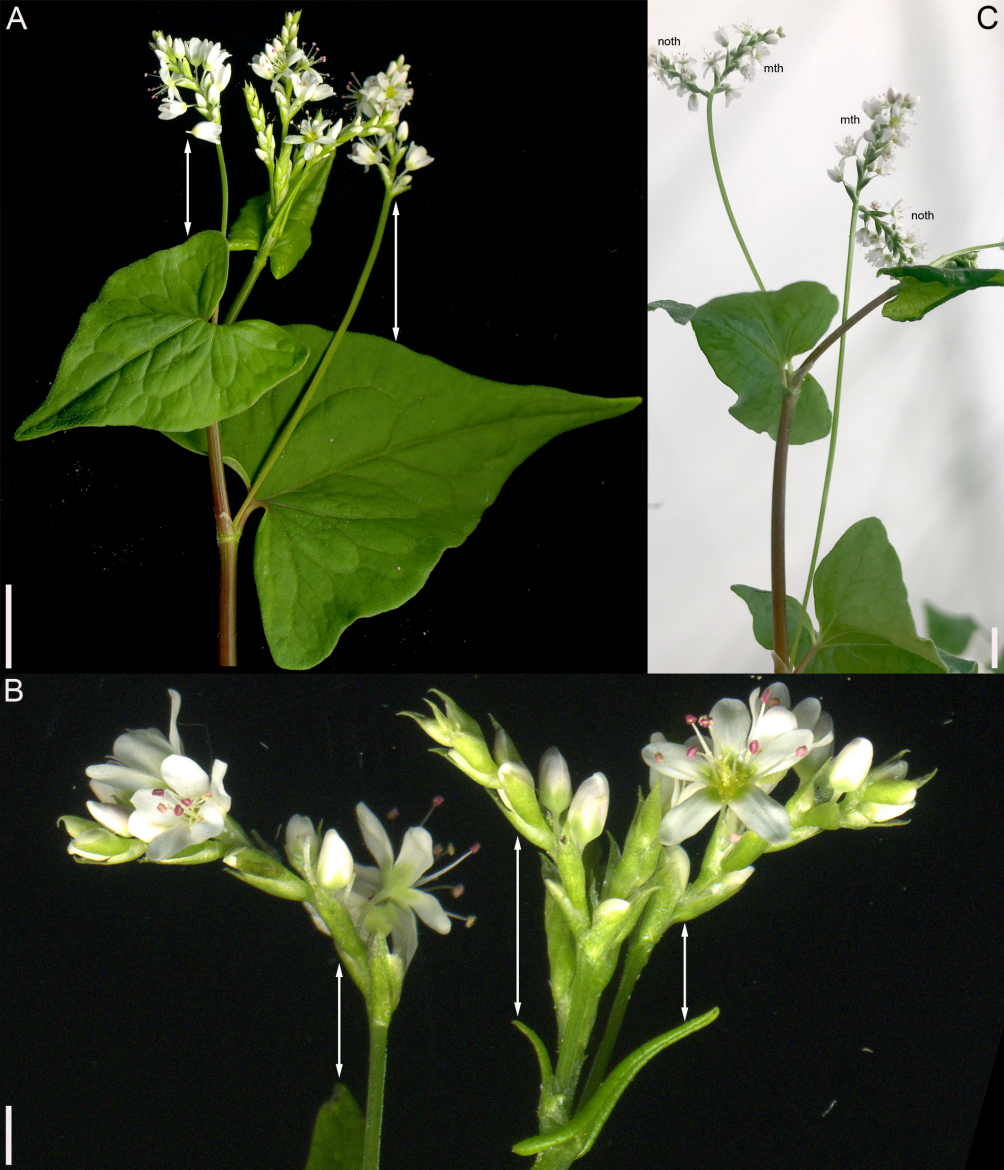
Inflorescence morphology in *Fagopyrum esculentum* subsp. *ancestrale*. **(A, B)** Upper part of inflorescence of typical morphology (see **Figure 5C**, for diagram). **(B)** Detail of inflorescence tip similar to that in **(A)** showing young lateral thyrses. Arrows in **(A, B)** show leaves subtending particular thyrses. **(C)** Unusual specimen with ‘double thyrses’ resembling those observed in *F tataricum* K17 and *F cymosum.* mth, main thyrse of a pair; noth, next order thyrse of a pair. Scale bars = 1 cm **(A, C)**, 2 mm **(B)**.

The species is currently classified into two subspecies (subsp. *ancestrale* and subsp. *esculentum*), the former comprising wild accessions from Southeast Asia and the latter cultivated accessions. In plants of subsp. *ancestrale* (**Figures 5C**, **9A, B**), the main axis of inflorescence bears foliage leaves and never terminates in a thyrse. The terminal flowering unit (dotted blue line in **Figure 5C**) contains numerous sequentially developing lateral thyrses. Paracladia of the first and second order develop similar flowering units. The uppermost paracladia of the first order approach the size of the terminal flowering unit (**Figure 5C**) whereas the proximal paracladia (not shown in **Figure 5C**) are smaller, but still well-developed. Paracladia develop even in the axils of the cotyledons. A few of the many individuals examined of subsp. *ancestrale* possessed flowering units with ‘double thyrses’ in the axils of some foliage leaves (**Figure 9C**). The same individuals also possessed simple lateral thyrses in axils of other foliage leaves. The ‘double thyrses’ exactly repeated branching patterns found in *F. cymosum* (**Figure 5E**, lowermost branch) and *F. tataricum* K17 (**Figure 5A**, upper branches). In each pair of thyrses, one belongs to the next order axis and develops in the axil of a scale-like phyllome. In contrast to K17 and other accessions of *F. tataricum*, flowering units of *F. esculentum* subsp. *ancestrale* never developed a terminal thyrse. Instead, they continued initiation and development of lateral thyrses even after four months of flowering.

**Figure 10:**
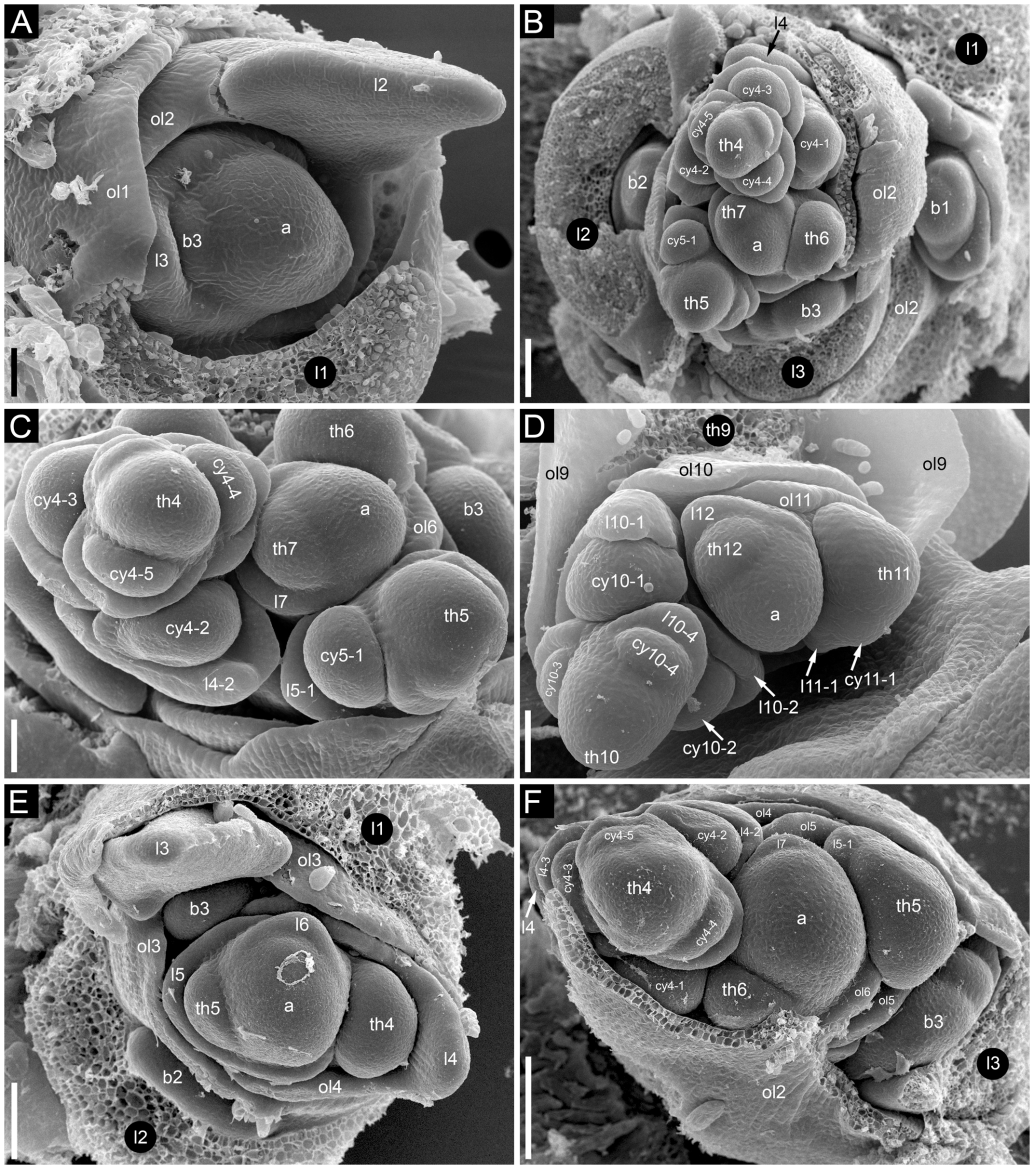
Developmental morphology of cultivars of *Fagopyrum esculentum* subsp. *esculentum* with indeterminate growth (SEM). In all instances, no terminal thyrse is produced. **(A–D)** cv. Batyr. **(E, F)** cv. Chatyr Tau. **(A)** Top view of young main axis with cotyledons and the first proper leaf removed. **(B)** Subsequent developmental stage, three first leaves are removed. Leaf 4 and subsequent leaves have axillary thyrses. **(C)** The same specimen as in **(B)** seen from another angle. **(D)** Main apex still producing lateral thyrses at a late developmental stage. **(E)** Top view of main axis with leaves 1 and 2 removed. **(F)** Top view of main axis with leaves 1–3 removed. In both **(D)** and **(E)**, the first thyrse occurs in the axil of leaf 4. a, apex of the main axis; b, branch apex (these branches will develop as paracladia); cy, cyme; l, leaf (foliage leaf or cyme-subtending bract); ol, ocrea of leaf; th, thyrse apex (or place of removed thyrse – th9 in **D**). Leaves of the main axis are numbered (cotyledons not counted). Leaves of lateral axes have double numbers, the first one being the number of the main axis leaf subtending the branch. Numbers of all axillary structures are those of their subtending leaves. Scale bars = 50 µm **(A, C, D)**, 100 µm **(B, E, F)**.

In subsp. *esculentum*, indeterminate cultivars Chatyr Tau and Batyr (**Figure 10**) possessed inflorescences similar to those in subsp. *ancestrale* (**Figure 5C**), though the flowering units contained fewer thyrses. ‘Double thyrses’ were not found.

**Figure 11:**
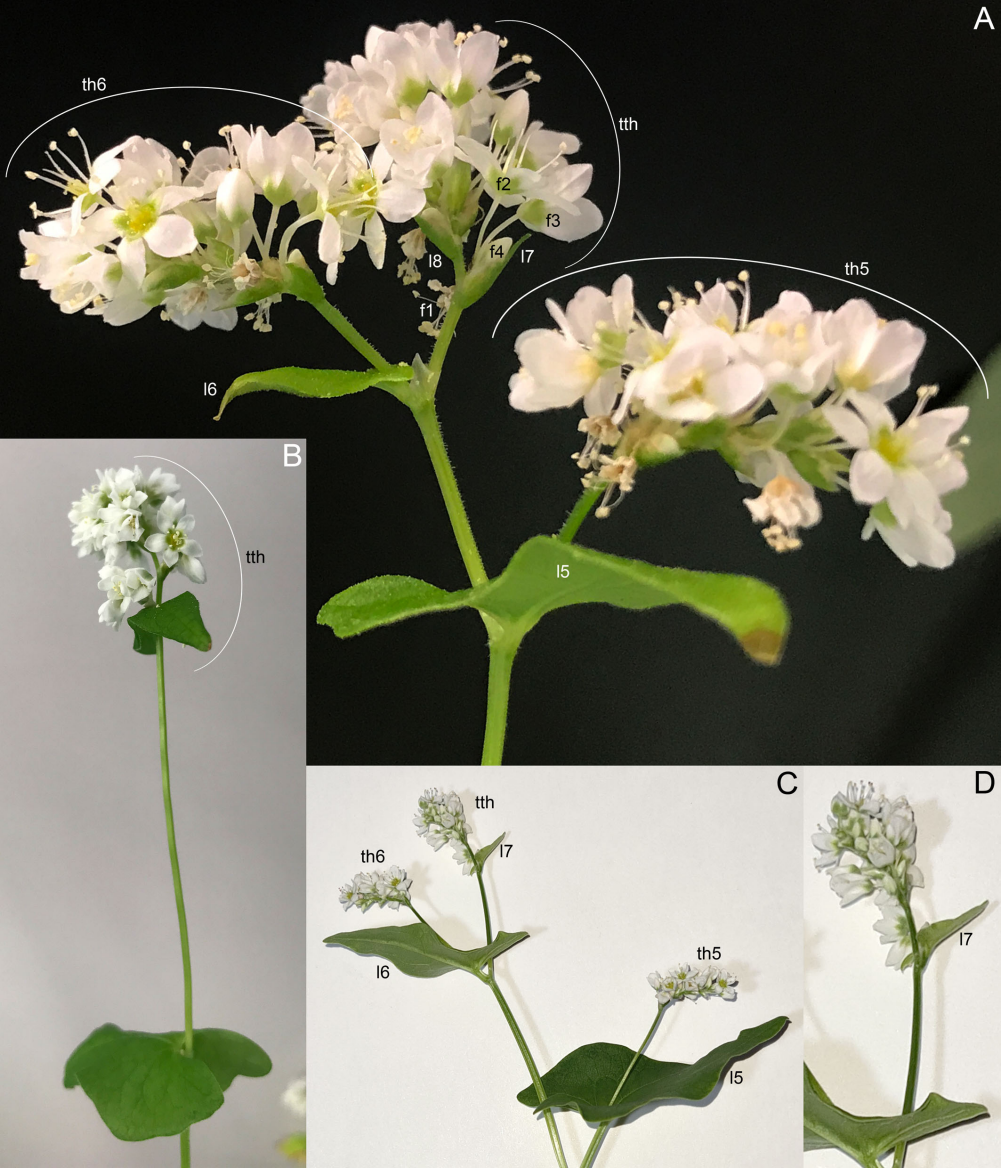
Inflorescence morphology of cultivars of *Fagopyrum esculentum* subsp. *esculentum* with determinate growth. **(A, B)** cv. Dasha. **(A)** Plant with a terminal and two lateral thyrses situated in axils of leaves 5 and 6 of the main axis. Leaf 7 is scale-like and subtends the first cyme of the terminal thyrse. **(B)** Plant with the entire inflorescence composed of a terminal thyrse. The first cyme of the terminal thyrse is subtended by a foliage leaf. **(C, D)** cv. Temp. Plant with a terminal and two lateral thyrses situated in axils of leaves 5 and 6 of the main axis. Leaf 7 is a foliage leaf and subtends the first cyme of the terminal thyrse. **(D)** is a detail of **(C)** showing the terminal thyrse. f1, f2, f3, f4, successive flowers of the first cyme of the terminal thyrse, f1 is postanthetic, unfertilized, f4 is a bud; l5, l6, l7, l8, phyllomes of the main axis counted starting from the first leaf above the cotyledons; th6, th7, thyrses in axils of leaves 6 and 7; tth, terminal thyrse.

**Figure 12:**
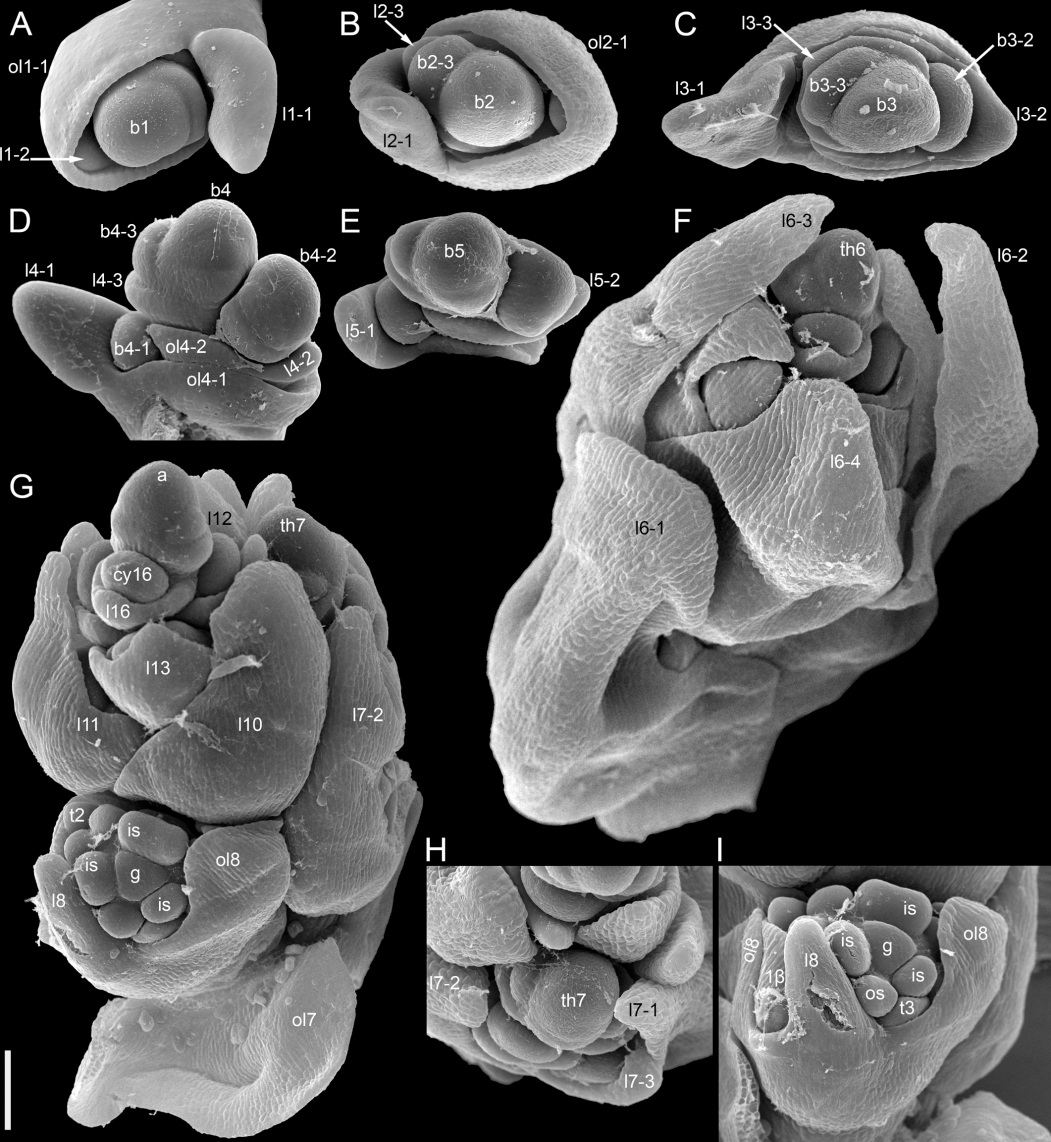
Dissected young plant of *Fagopyrum esculentum* subsp. *esculentum* cv. Dozhdik with determinate growth pattern (SEM). See **Figure 5**, for its diagram. The plate shows the terminal thyrse and branches developing in axils of all foliage leaves of the main axis except cotyledons. **(A)** Branch in the axil of the first leaf. Similar branches were found in axils of the cotyledons. **(B–E)** Branches in axils of the second, third, fourth and fifth leaf, respectively. **(F)** Lateral thyrse in axil of the leaf 6. **(G)** The terminal thyrse and a lateral thyrse in axil of the leaf 7 (right). **(H)** Apex of the thyrse in the axil of the leaf 7. **(I)** The first cyme of the terminal thyrse subtended by leaf 8 of the main axis. The leaf 8 has a reduced lamina. The first flower of the cyme is visible. It is at developmental stage 7 (as in **Figures 3G–I**). 1β, β-prophyll of the first flower of a cyme (see **Figure 3**); a, apex of the main axis; b, branch apex (where can be precisely identified, these branches are paracladia); cy, cyme; g, gynoecium; is, inner whorl stamens; l, leaf (foliage leaf or cyme-subtending bract); ol, ocrea of leaf; os, outer whorl stamens; th, thyrse apex; t2, t3, tepals 2 and 3 (see **Figure 3**). Leaves of the main axis are numbered (cotyledons not counted). Leaves of lateral axes have double numbers, the first one being the number of the main axis leaf subtending the branch. Numbers of all axillary structures are those of their subtending leaves. Scale bar (common to all images) = 100 µm.

**Figure 13:**
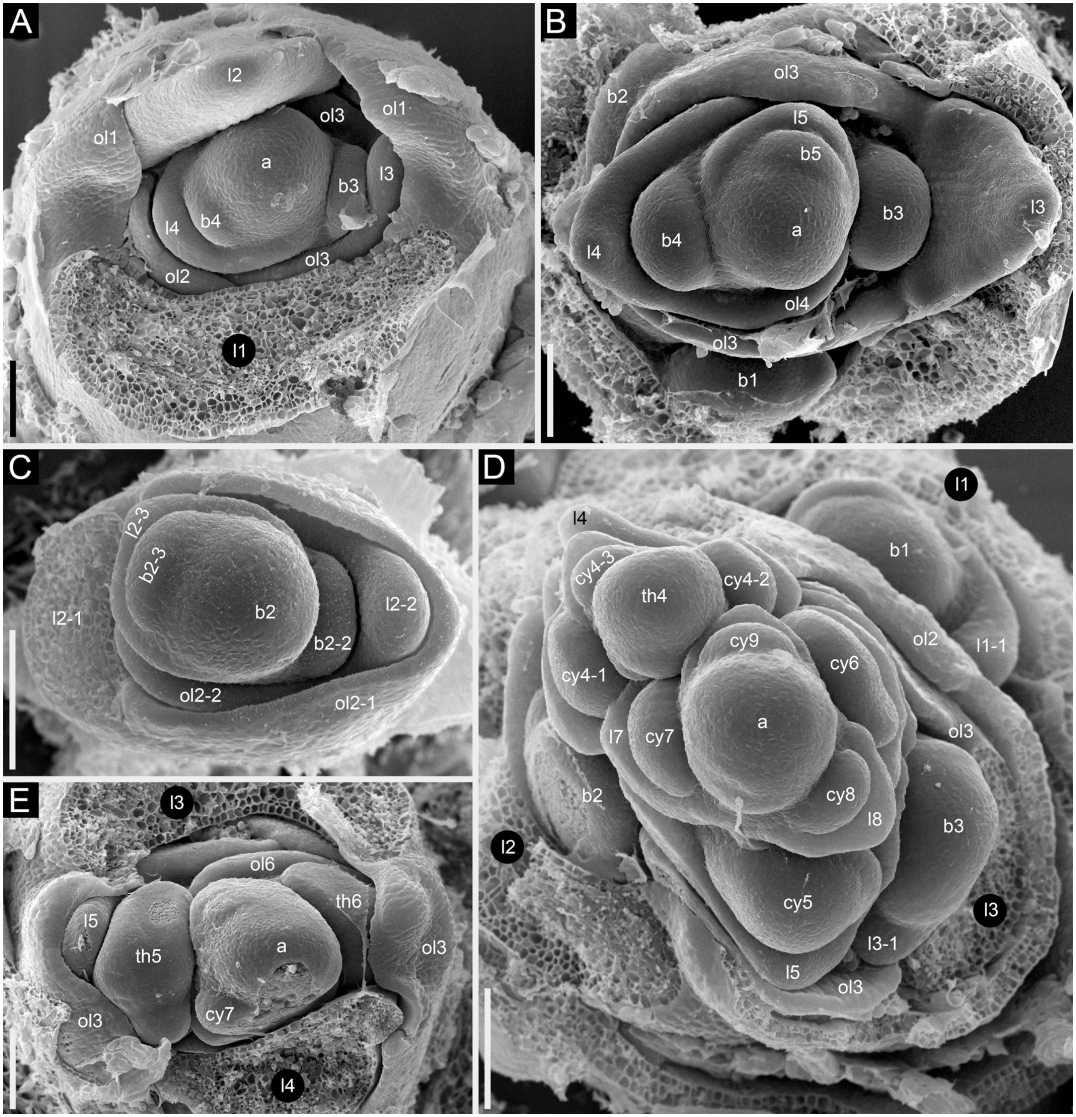
Developmental morphology of cultivars of *Fagopyrum esculentum* subsp. *esculentum* with determinate growth (SEM). **(A–C)** cv. Dozhdik. **(A)** Top view of young main axis with cotyledons and the first proper leaf removed. Branches in axils of leaves 3 and 4 visible here have a potential to develop into branched paracladia. **(B)** Top view of young main axis with cotyledons and two first proper leaves removed. Branches in axils of leaves 1 and 2 have a potential to develop into branched paracladia; the nature of other branches yet cannot be identified. **(C)** Top view of young paracladium developing in axil of leaf 2 of the main axis (adaxial side bottom). **(D, E)** cv. Temp. **(D)** Top view of young main axis with cotyledons and four first proper leaves removed. Branches in axils of leaves 1-3 have the potential to develop into branched paracladia. **(E)** Top view of young main axis with cotyledons and three first proper leaves removed. a, apex of the main axis; b, branch apex; cy, cyme; l, leaf (foliage leaf or cyme-subtending bract); ol, ocrea of leaf; th, thyrse apex. Leaves of the main axis are numbered (cotyledons not counted). Leaves of lateral axes have double numbers, the first one being the number of the main axis leaf subtending the branch. Numbers of all axillary structures are those of their subtending leaves. Scale bars = 100 µm **(A–E)**.

Determinate cultivars Dozhdik, Temp and Dasha (**Figures 5D**, **11**–**13**) possessed terminal thyrses in the axes of all branching orders. In most instances, their flowering units contained 1–3 lateral thyrses situated in the axils of the foliage leaves and a terminal thyrse. Only subtle differences in the timing of anthesis could be found between the terminal and lateral thyrses of the flowering unit. In extreme cases, the entire flowering unit was composed of a terminal thyrse (**Figure 11B**). The lowermost cyme-subtending phyllome of the terminal thyrse was often differentiated as a foliage leaf with a well-differentiated (**Figures 11B–D**) or much reduced lamina (**Figures 12G, I**). In other instances, all cyme-subtending phyllomes, including the first one, were scale-like (**Figure 11A**). Paracladia with flowering units repeating the branching pattern of the terminal flowering unit were much delayed in development.

#### Fagopyrum homotropicum

Inflorescence morphology is of the same pattern as in indeterminate plants of *F. esculentum* (**Supplementary Figure 2**). There is no terminal thyrse. ‘Double thyrses’ were not found. Plants illustrated here lack paracladia and all thyrses belong to the terminal flowering unit.

#### Developmental patterns and phyllotaxis

Phyllotaxis was spiral, at least in the distal parts of the shoots. When a terminal thyrse was present in *F. tataricum* and *F. esculentum*, the phyllotaxis spiral of foliage leaves of the main axis directly continued into the spiral of cyme-subtending phyllomes (**Figures 8**, **12**). The uppermost foliage leaves were at immature developmental stages by the time when their axillary thyrses or thyrse complexes commenced to anthesis (**Figures 6B, C**, **8**, **9B**). In extreme cases, the lamina was filiform and much shorter than the leaf ocrea, but it was always present. The phyllotaxis of the lateral thyrses was spiral from inception. The first and second cyme-subtending bracts were always in transversal positions. Their axillary cymes were always mirror-shaped relative to each other. The first flowers of these cymes were at a maximum distance from the subtending leaf of the thyrse. The third and fifth cyme-subtending bracts were located on the anterior side of the thyrse and the fourth bract was on the posterior side.

**Figure 14:**
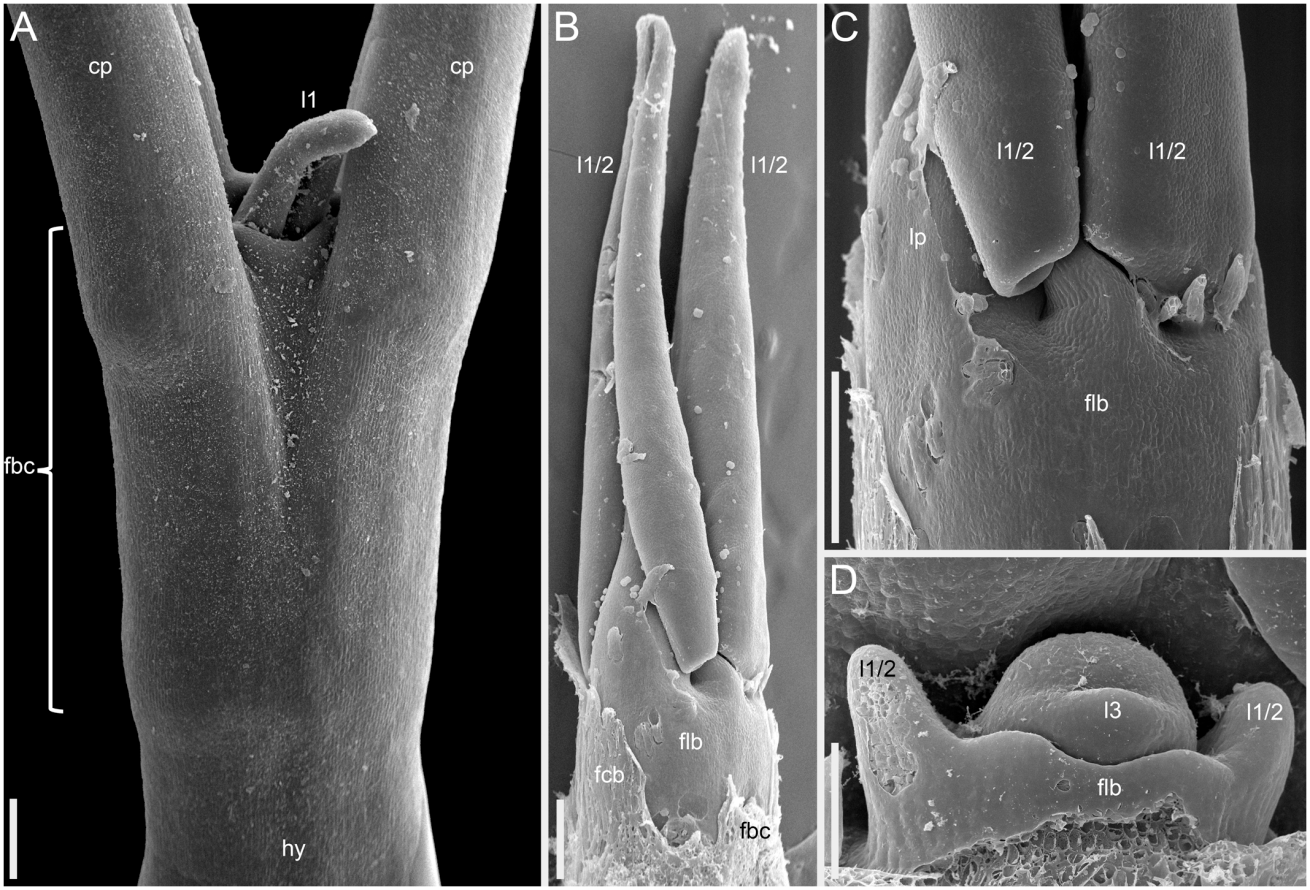
Instances of congenital fusion of leaves in *Fagopyrum esculentum* subsp. *esculentum* (SEM). **(A)** cv. Dozhdik. Fused bases of cotyledons. Developmental stage similar to that in **Figure 12**. **(B, C)** cv. Temp. An unusual instance of basal fusion between two first leaves of the main axis. **(C)** is the same sample as **(B)** viewed from another angle. **(D)** cv. Temp. An unusual instance of basal fusion between two first leaves of a paracladium. cp, cotyledon petiole; fbc, fused bases of cotyledons (largely removed in **B**); flb, fused leaf bases; hy, hypocotyl; l1, the first leaf above the cotyledons; l1/2, two first leaves above the cotyledons **(B, C)** or two first leaves of a paracladium **(D)**; l3, third leaf; lp, leaf petiole. Scale bars = 300 µm **(A–C)**, 100 µm **(D)**.

More developmental details were obtained for *F. esculentum* (**Figures 10**, **12**–**14**). The two cotyledons are basally united to form a sheathing tube. By the time the tip of the first leaf had emerged from the orifice of the cotyledonary tube (**Figure 14A**), several structures were already initiated: all foliage leaves of the main axis, all thyrses of the terminal flowering unit (at least in determinate cultivars) and the first flowers. Leaves 1 and 2 above the cotyledons were often at a divergence angle of about 180° from each other and 90° from the cotyledons. With subsequent leaves (or already from leaves 1 and 2), a spiral approaching a Fibonacci pattern was established. In exceptional instances, leaves 1 and 2 belonged to the same node and were basally united to form a sheathing tube (**Figure 14B**). In these instances, the first leaf pair was decussately arranged with respect to the cotyledons. Developmental patterns of indeterminate and determinate cultivars showed no pronounced differences until initiation of the terminal thyrse in determinate cultivars. Branching took place in the axils of all leaves, including the cotyledons. Branches below the first lateral thyrse potentially develop as paracladia, each terminated in a flowering unit. However, they can be arrested early in development without producing any branch visible without a dissection and microscopic observations in anthetic plants. The upper paracladia along the main axis generally show greater development than lower paracladia (**Figures 12A-E**), but still there is a major developmental gap between the uppermost paracladium (**Figure 12E**) and the lowermost lateral thyrse of the terminal flowering unit (**Figure 12G**). In paracladia, phyllotaxis always starts with two transversally spaced foliage leaves. In rare instances, these two leaves belong to the same node and fuse to form a common sheathing base (**Figure 14D**). The third leaf is always on the anterior side of a paracladium.

### Inflorescence morphology of *Fagopyrum urophyllum* (Urophyllum group)

**Figure 15:**
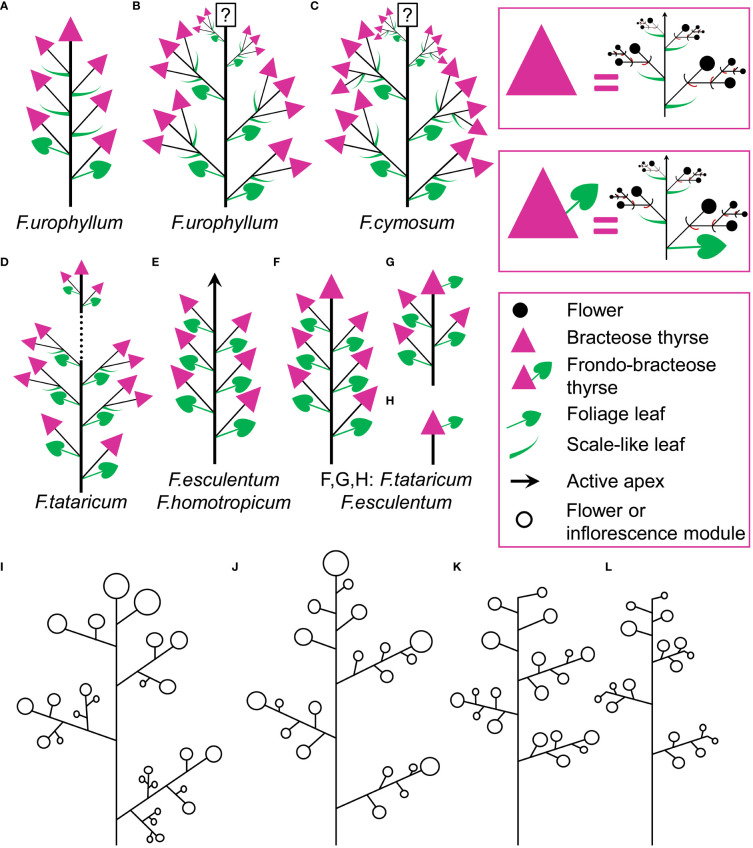
**(A–H)** Diversity of flowering units in *Fagopyrum* species studied here. Diagrams are simplified with respect to variation in number of branches on the main axis. Questions marks denote the absence of data on the latest developmental stages. **(I–L)** Typical scenario of angiosperm inflorescence evolution (from I to L) proposed in the framework of the so-called Pseudocycle Concept (modified from Kusnetzova, 1988). Circles may represent flowers or more complex aggregations of flowers (e.g. thyrses in Polygonaceae: Yurtseva, 2006). Although the Pseudocycle Concept is problematic in many respects, especially in the proposed unidirectional nature of its transformation series, the branching patterns included in the diagram appear to represent some ‘privileged’ types that tend to occur more commonly than other types among angiosperms, possibly for reasons of physiological regulation of inflorescence development.

The basic inflorescence module of *F. urophyllum* is a bracteose thyrse. Some specimens in collections of *F. urophyllum* possess inflorescences of the same type as described above for *F. cymosum* (for example, BNU 0026870, BNU 0026869, PE 01859341, PE 01859340, see https://www.cvh.ac.cn/). The main axis apparently has foliage leaves only (frondose structure), though the uppermost leaves are retarded in development. Structures in the axils of these leaves are groups of two or three thyrses. Branching on these second-order axes takes place in the axils of scale-like leaves (bracteose structure). The main inflorescence axis lacks a terminal thyrse, at least at developmental stages available for investigation, but the second-order inflorescence axes always possess a terminal thyrse (**Figure 15B**). The accession of *F. urophyllum* grown for the present study (**Supplementary Figure 3**) was similar to that described above, but tended to produce groups of more than three bracteose thyrses in the axils of the foliage leaves of the main axis. Branching in these groups of thyrses was either bracteose or frondo-bracteose.

Many herbarium specimens of *F. urophyllum* possess inflorescences with the axis terminated by a thyrse (**Figure 15A**). There are also lateral thyrses situated in the axils of the leaves of the main axis below the terminal thyrse. Lower lateral thyrses develop in the axils of foliage leaves, but upper ones are subtended by scale-like leaves (frondo-bracteose inflorescence structure). Third-order thyrses are normally absent. The terminal thyrse is usually longer than the uppermost lateral thyrse and equals the lower lateral thyrses (for example, PE 01062852, https://www.cvh.ac.cn/). Rarely, the terminal thyrse is short and, in contrast with most other specimens, anthesis is delayed in terminal and uppermost lateral thyrses as compared to the long lower lateral thyrses (PE 01859339). The terminal and lateral thyrses form what can be interpreted as flowering unit. Some specimens show lateral units of this sort that can be interpreted as developing on paracladia (for example, there is one paracladium in PE 00167717). Since *F. urophyllum* is a perennial and often robust plant, the complete branching pattern not always can be inferred from the herbarium material. Observations in nature are necessary to obtain a complete picture.

## Discussion

### The prophyllar sheath in *Fagopyrum* cymes is a fusion product of two prophylls

In cymes of *Fagopyrum* (tribe Fagopyreae) and many other Polygonaceae (members of tribes Polygoneae and Persicarieae, Sanchez et al., 2011), our data support the hypothesis that the bilobed sheath associated with each flower is composed of two congenitally fused prophylls, one of which subtends the next-order flower (Payer, 1857; Gross, 1913; Bauer, 1922; Galle, 1977; Ronse De Craene, 2022). This issue is far from trivial. The two prophylls (α and β) are most likely initiated as two distinct primordia, but their common sheathing part becomes conspicuous at very early developmental stages (Sattler, 1973). Photographs provided by Sattler (1973) are not fully convincing in the free nature of the two prophylls at their initiation, because their common tubular part could be hidden by the flower-subtending bract and main inflorescence axis. In a detailed study of *Bistorta officinalis* Delarbre (*=Polygonum bistorta* L.), Schumann (1890) disagreed with the double nature of the prophyllar sheath at its initiation and suggested that it is formed by a single prophyll. He contended that in rare instances when two prophylls are formed, they are free from each other from initiation, each subtending a next-order flower. He criticized Payer’s (1857) illustrations of early developmental stages in *Fagopyrum cymosum*, and suggested the occurrence of a single phyllome that forms the prophyllar sheath. Our **Figure 2C** shows convincing evidence of a developmental stage preceding the formation of the tubular sheath encircling the floral apex, at least on its adaxial side. Note however that a shallow abaxial connection can be already recognized between the two lobes (α and β) in a rather young developmental stage shown in **Figure 2E**.

In theory, initiation as two free primordia, taken in isolation from all other arguments, cannot be regarded as a necessary or sufficient condition of recognition of two congenitally united organs. In so-called early congenital fusion, free parts of united organs become visible after the initiation of their common part (Sokoloff et al., 2018). There are even cases when one and the same organ initiates as two distinct primordia. For example, in the flower of *Groenlandia densa* (L.) Fourr. (Potamogetonaceae), each lateral stamen arises as two separate primordia, whereas each median stamen initiates as a single primordium (Posluszny and Sattler, 1973).

Yurtseva (2006) re-interpreted the two lobes of the prophyllar sheath in cymes of Fagopyreae, Polygoneae and Persicarieae as two stipules of one and the same prophyll. Indeed, the ocrea of Polygonaceae can be viewed as a fusion-product of the two stipules and in the absence of a leaf blade one could easily imagine a sheathing phyllome composed of a pair of stipules. In contrast with Schumann (1890); Yurtseva (2006) concluded that the next-order flower is not subtended by the prophyll, but its subtending phyllome is completely suppressed. According to this interpretation, the single bilobed prophyll has an adaxial position relative to the flower-subtending bract (which is a cyme-subtending bract in the case of the first flower of a cyme). The two lobes (inferred stipules of the prophyll) are located in almost transversal positions (Yurtseva, 2006). The empirically observed position of the next-order flower is towards the abaxial (rather than adaxial) side of the first flower (Sattler, 1973; Yurtseva, 2006; present study). This represents the primary reason for not interpreting the next-order flower as occurring in the axil of a reportedly adaxial prophyll (Yurtseva, 2006).

Sattler’s (1973) diagram shows the two lobes of the prophyllar sheath as symmetrically arranged in transversal positions. Therefore, the next-order flower is out of the median of the β-lobe in Sattler’s diagram. Our data on the earliest developmental stages reveal differences in the shape and position of the lobes. The β-lobe is shifted towards the abaxial side rather than being strictly transversal; it is also wider than the α-lobe (**Figure 2E**). Some images taken at the mid stages of flower development still capture apparent differences in shape and position of the α- and β-lobe (for example, **Figure 2L**). Therefore, if the α- and β-lobes are assumed as two distinct prophylls, the position of the next-order flower can be safely interpreted as axillary with respect to the β-prophyll. Late development of the β-lobe is somewhat one-sided, so that its summit acquires a transversal position that fits Sattler’s diagram. Furthermore, our data on branching in the paracladia of *F. esculentum* provide examples of axillary bud displacement relative to the midline of its subtending leaf (for example, b2-3 and l2-3 in **Figure 13C**). An important argument against interpretation of the prophyllar sheath as formed by a single adaxial prophyll is the absence of adaxial prophylls in any other instances of branching in *Fagopyrum*. The two first phyllomes of all paracladia and the lateral thyrses are always in transversal positions. Therefore, it is logical to assume a transversal (rather than adaxial) position of the prophylls in the cymes.

Our interpretation of the prophyllar sheath as two fused prophylls agrees with other examples of pairwise leaf fusion in *Fagopyrum*. The most obvious example is the formation of the cotyledonary tube (**Figure 14A**). In all instances when we observed occasional whorled (opposite) leaf arrangement of foliage leaves in *Fagopyrum*, these leaves were congenitally united to form a sheathing tube (**Figures 14B, C**: at the first node of the main axis above the cotyledons; **Figure 14D**: at the first node of a lateral axis).

Plants of the *tlb* mutant of *F. esculentum* sometimes develop two free phyllomes associated with each flower, of which one phyllome clearly subtends a next-order flower (Fesenko et al., 2005; Yurtseva, 2006; Logacheva et al., 2008; present study). Yurtseva (2006) interpreted the sporadic occurrence of the two phyllomes as evidence for regain of the reportedly suppressed second prophyll that subtends the next-order flower. Our data show that the two occasionally free phyllomes in the *tlb* mutant are homologous to the two lobes of the prophyllar sheath found in wild-type plants. The homology is supported by the conserved relative positions of the two phyllomes/lobes relative to tepals 1–5 of the first-order flower of the cyme. Even more important, our data show a series of transitional forms between entirely free and strongly fused prophylls, including the condition of one-sided fusion (**Figures 4E, F**). The main morphogenetic effect of the *tlb* mutation is the acquisition of tepal-like features by the prophyllar sheath (Fesenko et al., 2005; Logacheva et al., 2008). Tepals of *F. esculentum* differ from prophylls in the occurrence of epidermal papillae, white or pink colouring and a narrow base. Tepals of *Fagopyrum* are largely free from each other, although located at the same level on the receptacle. The tendency for splitting of the prophyllar sheath into two distinct structures should be viewed as a manifestation of its tepal-like features. Wild-type plants of *F. esculentum* have a long internode (pedicel) below the tepals, but no stalk below the prophyllar sheath. With the acquisition of tepal-like features, *tlb* plants tend to elongate the stem region below the prophyllar sheath and between the two prophylls. Sometimes a mosaic of features can be seen in mutant plants, including a situation when stem elongation takes place between two still fused prophylls, thus producing a distorted prophyllar sheath (**Figure 4H**). To summarize, the data on the *tlb* mutant support rather than contradict our preferred interpretation of the prophyllar sheath as congenitally fused α- and β-prophylls.

The occurrence of two transversal prophylls in cymes of Polygonaceae is entirely consistent with the presence of the same condition in the sister family, Plumbaginaceae (Ronse De Craene, 2022).

### Early flower development and the non-trivial position of the first-formed tepal

Flowers of the tribes Fagopyreae, most Polygoneae and Persicarieae possess five tepals arranged with quincuncial aestivation, in which two tepals are completely outside the other tepals in the floral bud, two are completely inside and one tepal is intermediate (Ronse De Craene, 2022). The intermediate tepal has one margin covered by the adjacent outer tepal and the other margin covering the adjacent inner tepal. Some other Polygonaceae, such as *Rumex* and *Rheum*, possess a monocot-like condition of three outer plus three inner tepals, so the morphological interpretation of flowers with five tepals, as in *Fagopyrum*, Polygoneae and Persicarieae, has been extensively discussed. Assuming that the condition in *Rumex* and *Rheum* is trimerous and two-whorled, flowers with five tepals could be regarded as two-whorled and intermediate between dimerous and trimerous (2½-merous, Yurtseva and Choob, 2005). Then the intermediate tepal could be interpreted as belonging partly to the outer and partly to the inner whorl. However, phylogenetic placement of Polygonaceae among taxa with commonly pentamerous flowers suggests that flowers of *Fagopyrum* are pentamerous and one-whorled with quincuncial aestivation, a condition found in many other eudicots belonging to various orders and families (Ronse De Craene, 2022).

Eudicots with quincuncial aestivation of tepals or sepals often display sequential organ initiation in the perianth or calyx that could be interpreted as a 2/5 spiral. Two outer organs are initiated first, followed by the intermediate organ and the inner organs are the last ones to be initiated. This pattern has been most commonly reported for members of Polygonaceae with five tepals (Payer, 1857; Bauer, 1922; Sattler, 1973; Galle, 1977), but not properly documented using SEM. Schumann (1890) highlighted technical difficulties in making observations on the sequence of floral organ initiation in Polygonaceae and emphasised the need for theory-neutral descriptions. He concluded that the sequence of tepal initiation in *Bistorta officinalis* and apparently other Polygonaceae does not follow a 2/5 spiral. According to his data, following initiation of the two outer tepals in transversal positions, the third tepal to initiate is the (inner) adaxial one, followed by the two abaxial tepals (see also Yurtseva, 2000). However, our data on *F. esculentum* as well as those of Sattler (1973) do not support the ideas of Schumann (1890). After initiation of the two transversal tepals, the third one is initiated in an abaxial and not adaxial position (Sattler, 1973; present study). Our data do not contradict the idea of tepal initiation along a 2/5 spiral, but we were unable to find precise evidence for sequential initiation of tepals 1 and 2 as well as tepals 3 and 4. We believe that the epi-illumination light microscopy photographs of Sattler (1973), like our SEM images, do not show a stage with only one tepal initiated and a stage with only four tepals initiated. Thus, our numbering of tepals is based partly on theoretical grounds. Tepal 1 appears to be larger than tepal 2 in **Figures 2F** and **2H**, so that their initiation in a very rapid sequence remains a plausible possibility.

Even though the initiation of the five outermost floral organs along a 2/5 spiral in *Fagopyrum* is shared with many other eudicots, the relative arrangement of the tepals and floral prophylls (bracteoles) in buckwheat and other Polygonaceae merits special attention. Eichler (1875) proposed that the arrangement of the outer floral organs (such as sepals) is to a large extent determined by the position of the floral prophylls. When five sepals (or tepals) have sequential patterning, the sequence should be initiated by the floral prophylls. The first sepal thus normally appears either in an adaxial-transversal or in an abaxial-transversal position, in both instances closer to the α-prophyll than to the β-prophyll (see **Figure 13** in Eichler, 1875). The first tepal of *Fagopyrum* is located in almost exactly the same radius as the α-prophyll, a condition that differs from the two possibilities predicted by Eichler (1875). Note that Engler’s (1875) diagrams of *Bistorta officinalis* and *Coccoloba nitida* Kunth (*C. guianensis* Meisn.) do not show a superposition of the α-prophyll and the first of the five tepals. In this respect, these diagrams differ from our diagram of *F. esculentum* and the diagram of *Persicaria lapathifolia* in Ronse De Craene (2022). Further studies will determine if any variation in tepal positions occurs in Polygonaceae.

The non-trivial superposed position of the first tepal and the α-prophyll could be related to the asymmetric position of the two prophylls in *Fagopyrum*, though the prophylls of *Persicaria* were illustrated as symmetric by Ronse De Craene (2022). Another peculiar feature of *Fagopyrum* and other Polygonaceae is the shape of the floral primordium and the young flower, which is pronouncedly elliptical rather than circular in outline. Some images even suggest a crescent-shaped outline of the young flower tightly packed between the cyme-subtending bract and the thyrse axis. Refined mathematical modelling of early flower development in Polygonaceae may shed new light on the problem, especially when considering aspects of pre-patterning as well as mechanical pressure and the peculiar shape of the floral apex (Choob and Yurtseva, 2007; Ronse De Craene, 2018; Bull-Hereñu et al., 2022).

In the framework of the inhibitory field theory, the superposed position of the α-prophyll and the first tepal could be conditioned by an inhibitory influence from the β-prophyll that much exceeds that of the α-prophyll. Such differences between the two prophylls would be intriguing, because they are located at the same level and fuse with each other to form the prophyllar sheath. In all other instances of pairwise phyllome fusion observed in the present study (between cotyledons or vegetative foliage leaves), no evidence was found for similar superposition with the next-formed organs. For example, in **Figure 14D**, the two first leaves of a shoot are basally united and the third leaf occupies a position between their radii. It seems that in contrast to the situation shown in **Figure 14D**, the α- and β-prophylls fuse despite their sequential pre-patterning.

According to Sattler (1973), immediately after inception of the fifth tepal primordium (or even simultaneously with it) a pair of outer-whorl stamen primordia is initiated opposite each of the first two tepal primordia in *F. esculentum*. According to our data, the first evidence of initiation of these stamens can be seen at even earlier stages, before initiation of the fourth and fifth tepal (**Figure 2J**). The timing of initiation of the outer stamens found in the present study agrees with observations in members of Polygoneae and Persicarieae (Galle, 1977). Leins and Erbar (2010) summarized other examples of asynchronous organ initiation in different floral sectors and highlighted the possible sectorial differentiation in activity of genetic regulatory networks determining organ identity.

### The concept of flowering unit offers a tool for inflorescence typology in *Fagopyrum*

Our data on inflorescence morphology in *Fagopyrum* fit well with the concept of flowering unit (Maresquelle, 1970; Sell, 1976; Kusnetzova, 1988; Acosta et al., 2009). As in other angiosperms with a pronounced flowering unit, in *Fagopyrum* the lower boundary of the terminal flowering unit is determined by an abrupt change in fate and speed of development of the axillary branches along the main axis. Lateral axes belonging to the terminal flowering unit show an acropetal or almost simultaneous development, whereas the paracladia situated below the terminal flowering unit are developmentally retarded and tend to have a basipetal sequence of flowering. It is remarkable that Fesenko et al. (2004), using another terminology, provided a diagram of plant architecture in buckwheat that perfectly fits the concept of the terminal flowering unit and paracladia of various orders bearing additional flowering units. Yurtseva (2006) and Kostina and Yurtseva (2021) used the concept of flowering unit to describe inflorescence diversity at the level of Polygonaceae and in the genus *Atraphaxis*.

As highlighted by Sell (1976), the flowering unit provides taxon-specific characters of flower arrangement and represents a simplest possible pattern of inflorescence structure in a given species. Indeed, the occurrence of paracladia is somewhat optional and partly determined by environmental conditions, whereas the terminal flowering unit does develop in any plant that has achieved transition to anthesis. Of course, the degree of development of the paracladia of the first and subsequent orders is not solely determined by the environment, but depends on an interplay with the genetic background. For example, the wild Common buckwheat (*F. esculentum* subsp. *ancestrale*) develops more paracladia than cultivars of *F. esculentum* subsp. *esculentum* in common garden experiments (Fesenko et al., 2004). Some cultivars tend to develop the terminal flowering unit only. The present study showed that paracladia do initiate in the axils of all foliage leaves below the terminal flowering unit, even if they are subsequently arrested in development.

Comparisons at the level of the flowering unit provide a clear summary of inflorescence diversity in *Fagopyrum*. The following characters can be proposed. 1. Presence (**Figures 15D, F–H**) or absence (**Figure 15E**) of a terminal thyrse. 2. Presence (**Figures 15A–G**) or absence (**Figure 15E**) of lateral thyrses. 3. Presence (**Figures 15B–D**) or absence (**Figures 15A, E–G**) of bracteose branching of the stalks of lateral thyrses. 4. Bracteose (**Figures 15A, D, F**) or frondo-bracteose (**Figures 15G, H**) structure of the terminal thyrse. 5. Frondose (**Figures 15B–G**) or frondo-bracteose (**Figure 15A**) structure of the entire flowering unit (terminal thyrse not considered).

### Terminal thyrse, axiality and determinate growth patterns

Our data show that the determinate growth pattern of cultivars Dozhdik, Temp and Dasha of *F. esculentum* is conditioned by the presence of the terminal thyrse in flowering units. Plants of *F. esculentum* with indeterminate growth (including those of ssp. *ancestrale*) as well as the closely related wild species *F. homotropicum* lack a terminal thyrse. These differences were already correctly described for *F. esculentum* by Fesenko (1968), though he incorrectly used the term ‘raceme’ for thyrse.

The present study highlights, apparently for the first time, important differences between wild accessions of *F. tataricum* and *F. esculentum*, with respect to the presence vs. absence of a terminal thyrse. Thus, our data suggest different evolutionary patterns in the two species. Selection towards fixation of the determinate growth pattern conditioned by the occurrence of a terminal thyrse took place in domesticated *F. esculentum*. In contrast, the cultivated accession of *F. tataricum* studied here (K17) is characterized by a delayed ontogenetic transition to terminal thyrse formation, compared with other accessions of this species. In theory, further heterochronic shifts in this direction may lead to permanent elimination of the terminal thyrse. It is tempting to suggest heritability of the striking differences in the timing of ontogenetic transition to terminal thyrse observed here among accessions of *F. tataricum*. Indeed, in a common garden experiment, at the stage when terminal thyrses were fully formed after 10-11 nodes of the flowering unit in C9119, no indication of their initiation was visible in K17. Furthermore, the ruderal accession Zhd001 initiated a terminal thyrse as early as starting from the third node above the cotyledons. Importantly, the occurrence of a terminal thyrse does not guarantee a determinate growth pattern in *F. tataricum*. The plant of *F. tataricum* C9119 illustrated in **Figure 7** has a terminal thyrse, but does not meet the conditions of the determinate growth pattern. Note that the fruits are shed in lower thyrses of the terminal flowering unit whereas the upper ones are still in anthesis and fruit development. The accession K17 is even more problematic in this respect because in our experiments it developed at least 19 nodes with lateral thyrses within the flowering unit before formation of the terminal thyrse.

The concept of determinate growth pattern is not equivalent to the concept of determinate inflorescences. The former is related to the applied problem of more synchronized flowering/fruit set and early cessation of inflorescence growth, whereas determinate inflorescences are defined morphologically as those bearing a terminal flower (Weberling, 1989). The basic inflorescence unit of Polygonaceae is a thyrse that by definition lacks a terminal flower (Endress, 2010). Thyrsoids (which differ in the presence of a terminal flower, Endress, 2010) are extremely rare in Polygonoideae (Yurtseva, 2006) and not recorded in *Fagopyrum*. Therefore, determinate inflorescences do not occur in this group. The presence versus absence of the terminal thyrse is related to the concept of axiality, which is a more general concept than that of determinate/indeterminate inflorescences (Weberling, 1989). Axiality measures the minimum number of axis orders formed before the axes terminate in flowers (Weberling, 1989). Thus, plants bearing a terminal thyrse are diaxial and those with only lateral thyrses are triaxial. Use of the concept of axiality much simplifies descriptions of branching patterns and allows large-scale comparisons. It can be employed even in angiosperm-wide data sets. Axiality has been successfully employed in genus-level studies of various angiosperms (Notov and Kusnetzova, 2004; Sokoloff et al., 2015). Interestingly, the phenotypic effects of mutants of *TFL* homologues in various angiosperms can be generalized as a decrease in the level of axiality. For example, wild-type *Arabidopsis* is diaxial, the *tfl* mutant is monoaxial; wild-type Pigeon Pea is 4-axial, the *Dt1* mutant is 3-axial (Bradley et al., 1997; Singer et al., 1999; Teeri et al., 2006; Sinjushin, 2013; Benlloch et al., 2015; Saxena et al., 2017).

As highlighted by Yurtseva (2006), thyrses of various Polygonaceae-Polygonoideae show either determinate or indeterminate growth patterns despite possessing the same basic groundplan of flower arrangement. Our study revealed that the terminal thyrse is extremely variable in its length and apparent duration of growth among examined material of *F. tataricum*. Some specimens had a long, frondo-bracteose terminal thyrse approaching an indeterminate condition. In *F. tataricum*, the morphological difference is striking between a morphotype with a long terminal frondo-bracteose thyrse and no lateral thyrses on the one hand, and one with a short terminal and many lateral thyrses, on the other. An environmental component in these differences should be considered, and the possibility of a trade-off between formation of lateral thyrses and lateral cymes, along with possible heritability of the observed differences (see Geber, 1990). One could suggest that cymes occurring in the axils of foliage leaves develop as a result of homeotic replacement of lateral thyrses, thus giving rise to a long frondo-bracteose terminal thyrse. However, testing this hypothesis is problematic. Another potential phenomenon is extension of the foliage leaf developmental program into the proximal region of the terminal thyrse. Indeed, the developmental conditions of the terminal thyrse differ from those of lateral thyrses in *F. tataricum* and *F. esculentum* because the terminal thyrse continues a shoot with foliage leaves. A tendency for foliage leaf development in the first node of the terminal thyrse was observed here in both *F. tataricum* and *F. esculentum*.

### The unusual inflorescence of *F. cymosum* and its reiteration in three other *Fagopyrum* species

Kusnetzova (1988) developed the earlier ideas of Maresquelle (1970); Sell (1976) and other authors on the possible patterns of evolutionary transformation of angiosperm inflorescences. These ideas, especially the unidirectional nature of the proposed transformations, are sensitive to criticism that is beyond the scope of the present paper. More recent studies proposed other ideas on inflorescence evolution (e.g. Claßen-Bockhoff and Bull-Hereñu, 2013; Stützel and Trovó, 2013). However, an important point is the occurrence of certain ‘privileged’ patterns (**Figures 15I–L**) of arrangement of individual flowers and flower aggregations that are repeated across various angiosperm groups (Kusnetzova, 1988). Interestingly, the pattern of thyrse arrangement described here for *Fagopyrum cymosum* does not apparently fit any of these patterns. Yurtseva (2006) did not mention this pattern while discussing the inflorescence diversity in Polygonaceae. Therefore, it merits special attention. *Fagopyrum cymosum* has complex lateral aggregations of thyrses and no terminal thyrse. The complexity of these lateral aggregations increases towards the distal part of the entire inflorescence.

Inflorescences essentially similar to those of *F. cymosum* (**Figure 15C**) are found, along with other patterns, in three other species of the genus, *F. urophyllum* (**Figure 15B**, **Supplementary Figure 3**), *F. tataricum* (**Figure 15D**, though with a delayed development of the terminal thyrse) and *F. esculentum* (**Figure 9C**). This re-iteration could represent an example of Vavilov’s (1922) Law of Homologous Series in variation. The inflorescence variation found in *F. urophyllum* is noteworthy because the two patterns (**Figures 15A, B**) are morphologically contrasting and as far as can be inferred from herbarium material strongly differ in their frequencies. One of the conditions found in *F. urophyllum* (**Figure 15A**), is interesting in its frondo-bracteose structure: distal lateral thyrses are located in the axils of the scale-like leaves of the main inflorescence axis. This structure differs from members of the Cymosum group of *Fagopyrum*, where the main axis has foliage leaves, at least until the terminal thyrse, if one is present (**Figures 15B–G**). Field observations on patterns of flower arrangement in various localities of *F. urophyllum* are needed to get a complete picture of diversity of this species, which consists of two phylogenetically distinct lineages (Kawasaki and Ohnishi, 2006). *Fagopyrum cymosum* (like *F. urophyllum*) is a perennial species and the only perennial member of the Cymosum group. Its phylogenetic position does not preclude a plesiomorphic condition for (some) its morphological traits, including inflorescence morphology. If *F. tataricum* is derived from *F. cymosum* (Yasui and Ohnishi, 1998; Yamane et al., 2003; Ohnishi, 2016), the pattern found in *F. cymosum* could be plesiomorphic for *F. tataricum*. However, given the occurrence of infraspecific variation and complex evolutionary patterns in *Fagopyrum* that involve polyploidy, use of conventional approaches of character evolution are problematic here. It is more appropriate to develop studies in evolutionary developmental genetics of buckwheat in the context of microevolution, speciation, and natural and artificial selection.

## Conclusions

We provide a formal classification of the early stages of flower development and define diagnostic characters that allow recognition of each stage. This classification will be important for further experimental studies of buckwheat. We confirm sequential tepal and stamen initiation with an overlap between these two processes in buckwheat, refine details of this phenomenon and illustrate it for the first time using SEM. Our study represents apparently the most complete SEM-based study of early flower development in Polygonaceae. The prophyllar sheath of *Fagopyrum* is composed of two fused prophylls. The relative positions of the floral prophylls and tepals in buckwheat and some other Polygonaceae is uncommon among eudicots and at least at first glance cannot be easily interpreted by the Inhibitory Field theory. Our data can be used for mathematical modelling of early flower development that should incorporate parameters such as the non-spherical shape of the floral apex and mechanical forces. Our investigation provides clear and simple characters of inflorescence morphology that can be readily formalized and will provide advances in the taxonomy, evolutionary biology and breeding of buckwheat.

Characters of inflorescence morphology can vary within species in *Fagopyrum*, and the same condition can be sometimes found in more than one species. At least in some instances, the variation is discrete; in the case of terminal thyrse development in Common buckwheat, the variation is determined by a single-gene mutation. Therefore, the conventional way to study character transformations against species trees is not appropriate in this case; it is further complicated by the occurrence of polyploidy and apparent reticulate evolution. Despite these limitations, the genus *Fagopyrum* offers an excellent opportunity for evo-devo studies related to inflorescence architecture. Our discovery of variation of inflorescence characters in self-pollinated species of *F. tataricum* offers a potential model system for the determination and molecular characterization of the genes responsible for this variation. Finally, we show that although the agriculturally important character, determinate growth pattern, is defined by the presence of a terminal thyrse in *F. esculentum*, in a wider context of the genus *Fagopyrum*, a terminal thyrse does not guarantee a more synchronized flowering pattern.

## Data availability statement

The original contributions presented in the study are included in the article/**Supplementary Material**. Further inquiries can be directed to the corresponding author.

## Author contributions

DS and ML designed the study. ML, AF and IF provided living plant material for the study. DS, RM, MR, AF, IF and ML made morphological observations and interpretations of inflorescence morphology. DS and RM conducted developmental studies in Moscow using scanning electron microscopy. DS, MR and PR studied and interpreted the reproductive development in the *tlb* mutant of *F. esculentum*. CF performed X-ray microtomography. All the authors reviewed drafts of the paper and approved the final version.

## Supplementary material

Supplementary Figure 1 Morphology of the ruderal accession of *Fagopyrum tataricum* (Zhd001). The plants were grown at Skolkovo Institute of Science and Technology in the same conditions as those of K17 and C9119 from a seed sample collected in a ruderal habitat near a railway in Moscow Province, Russia. Each plant has two cotyledons followed by two foliage leaves without obvious branches and a terminal frondo-bracteose thyrse.

Supplementary Figure 2 Inflorescence morphology in plants of *Fagopyrum homotropicum* grown at the All-Russia Research Institute of Grain Legumes and Groat Crops, Orel. **(A)** Two entire plants. All thryses are lateral and belong to the terminal flowering unit. Paracladia are not developed. **(B)** Top view of the flowering unit. **(C)** Side view of distal part of another flowering unit. All thyrses are lateral.

Supplementary Figure 3 Inflorescence morphology of plants of *Fagopyrum urophyllum* grown at the All-Russia Research Institute of Grain Legumes and Groat Crops, Orel. Left, most inflorescence branches possess three thyrses following the pattern illustrated in the Figure 15B, but there is a branch with four thyrses (arrowhead). Right, inflorescence with most branches bearing multiple thyrses. Note the occurrence of thyrses of three branching orders (resembling the pattern illustrated in the Figure 15C, but even more complex). Some inflorescence branches are frondo-bracteose (arrowheads).
