# Supplementary figures and images for: Reproductive development of common buckwheat (Fagopyrum esculentum Moench) and its wild relatives provides insights into their evolutionary biology
Source: Front Plant Sci. 2023 Jan 12;13:1081981. doi: 10.3389/fpls.2022.1081981 (PMC9877541; doi:10.3389/fpls.2022.1081981)

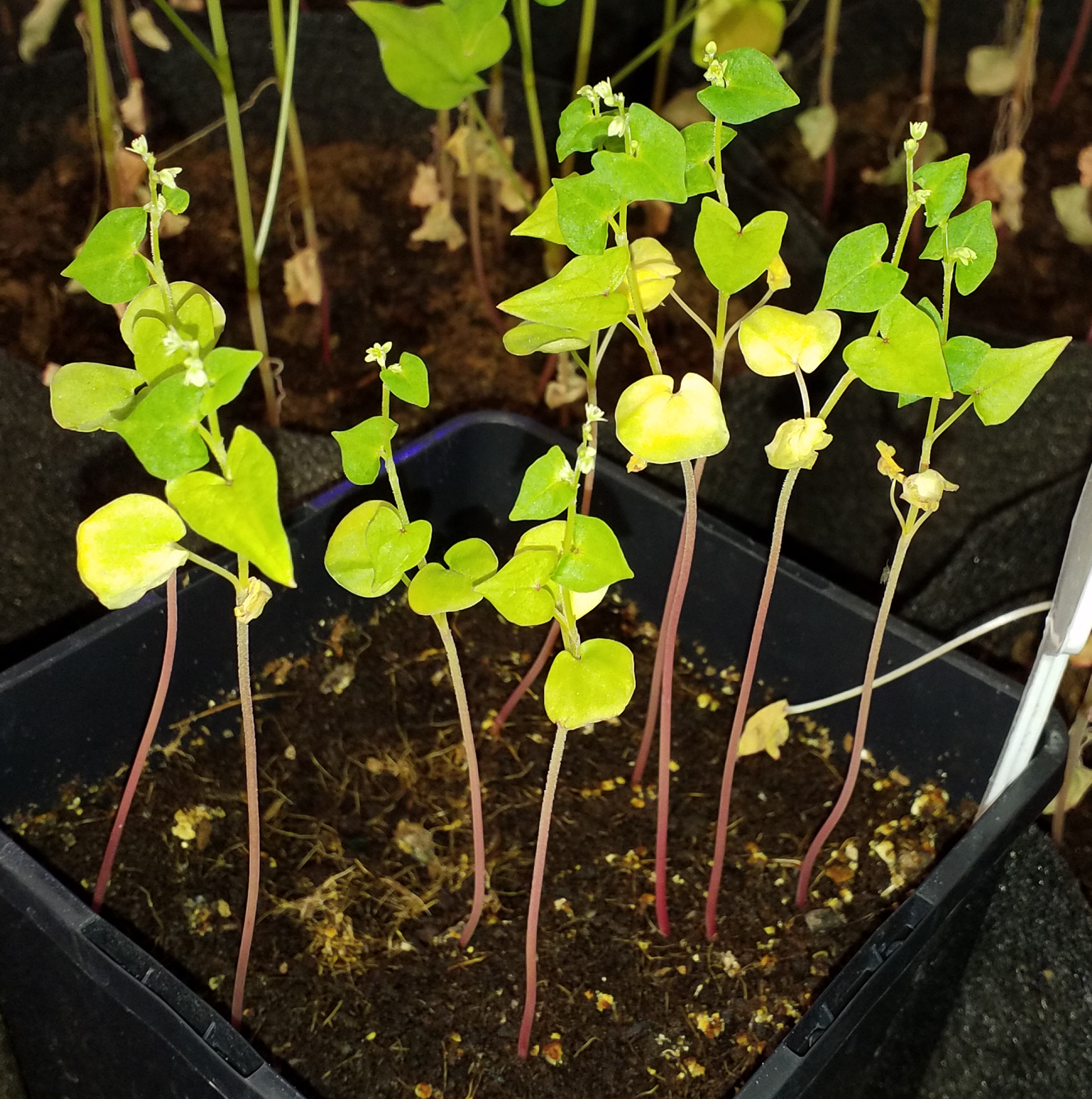

Supplement: Supplementary Figure 1 — Morphology of the ruderal accession of Fagopyrum tataricum (Zhd001). The plants were grown at Skolkovo Institute of Science and Technology in the same conditions as those of K17 and C9119 from a seed sample collected in a ruderal habitat near a railway in Moscow Province, Russia. Each plant has two cotyledons followed by two foliage leaves without obvious branches and a terminal frondo-bracteose thyrse. [file Image_1.tif]

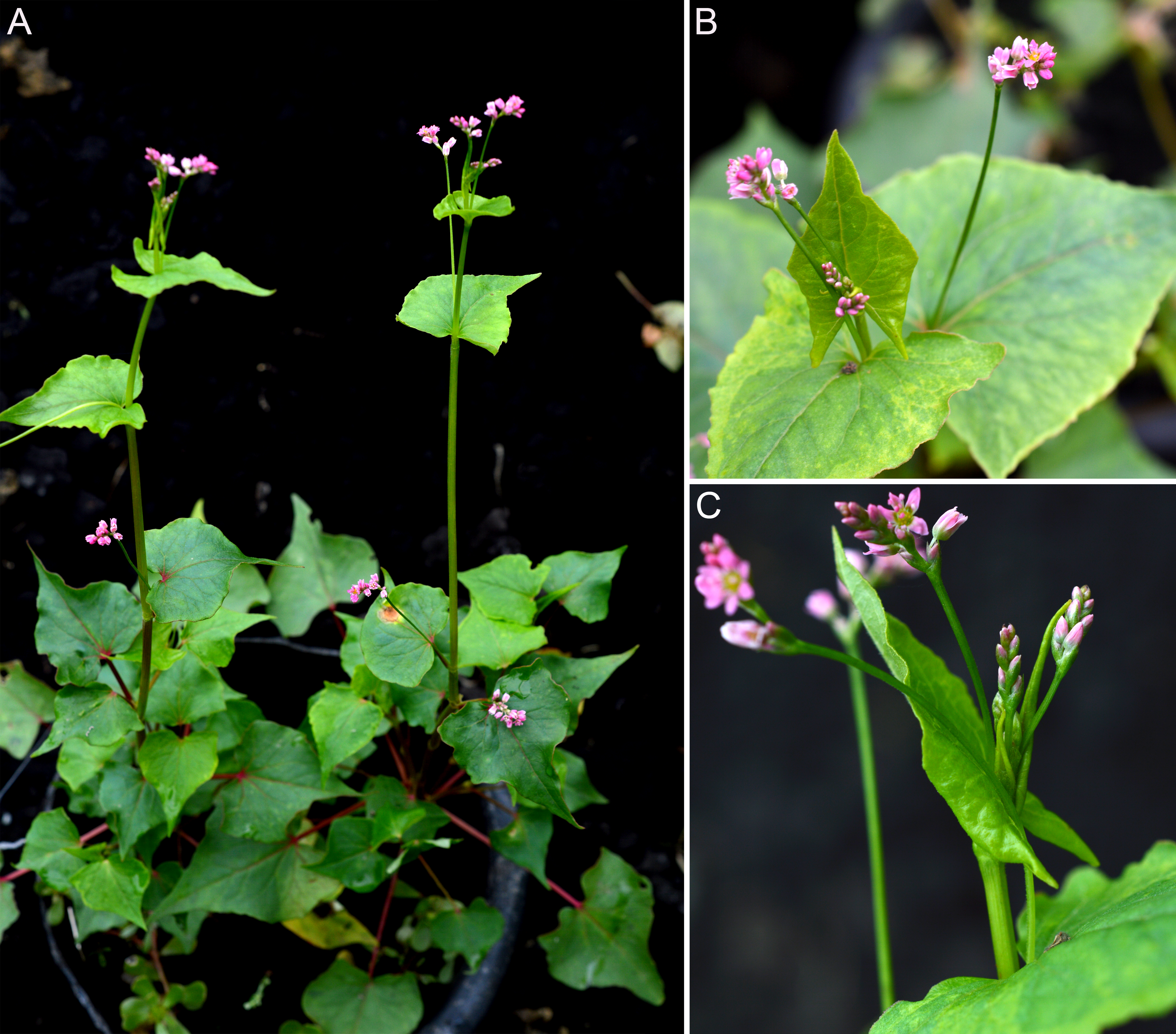

Supplement: Supplementary Figure 2 — Inflorescence morphology in plants of Fagopyrum homotropicum grown at the All-Russia Research Institute of Grain Legumes and Groat Crops, Orel. (A) Two entire plants. All thryses are lateral and belong to the terminal flowering unit. Paracladia are not developed. (B) Top view of the flowering unit. (C) Side view of distal part of another flowering unit. All thyrses are lateral. [file Image_2.tif]

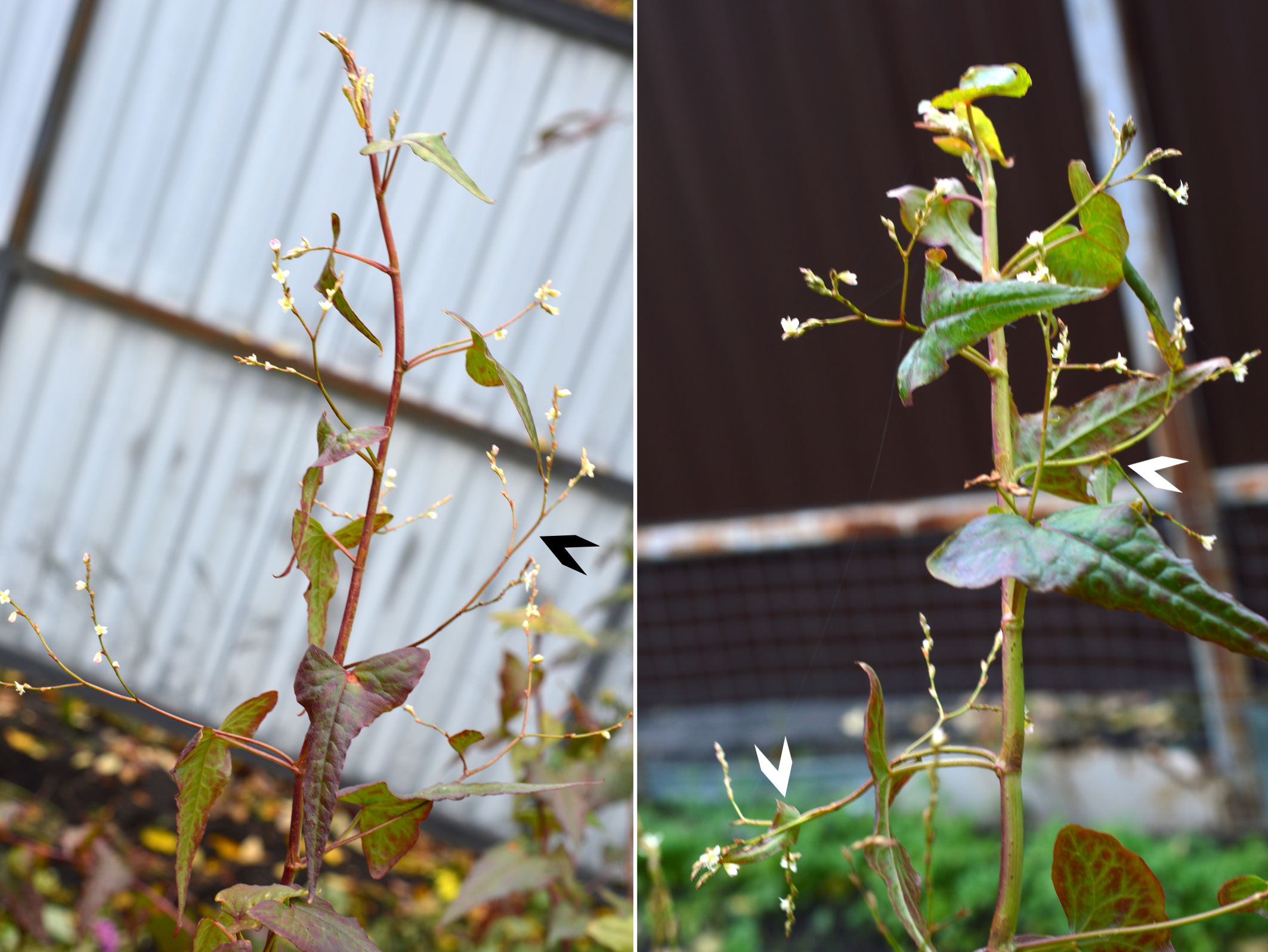

Supplement: Supplementary Figure 3 — Inflorescence morphology of plants of Fagopyrum urophyllum grown at the All-Russia Research Institute of Grain Legumes and Groat Crops, Orel. Left, most inflorescence branches possess three thyrses following the pattern illustrated in the Figure 15B, but there is a branch with four thyrses (arrowhead). Right, inflorescence with most branches bearing multiple thyrses. Note the occurrence of thyrses of three branching orders (resembling the pattern illustrated in the Figure 15C, but even more complex). Some inflorescence branches are frondo-bracteose (arrowheads). [file Image_3.tif]
